# Polypyrrole-Coated Microneedle Platform for Offline Electrochemical Detection of Interferon-Alpha in Interstitial Fluid

**DOI:** 10.1021/acsabm.5c01937

**Published:** 2026-02-11

**Authors:** Ana Carola Delavia Reis, Ana Cristina Honorato de Castro-Kochi, Jose Eduardo Ulloa Rojas, Dylan A. Chiaro, Suchismita Guha, Gavin M. King, Vivian L. de Oliveira, Daniele Ribeiro de Araujo, Giovana Radomille Tofoli, Mariano Romero, Dominique Mombrú, Wendel A. Alves

**Affiliations:** † Center for Natural and Human Sciences, 74362 Federal University of ABC, Santo André, São Paulo 09210-580, Brazil; ‡ School of Biomedical Engineering, Einstein Hospital Israelita, São Paulo, São Paulo 05521-200, Brazil; § Department of Physics and Astronomy, 14716 University of Missouri, Columbia, Missouri 6521, United States; ∥ Laboratory of Immunology, INCOR, HCFMUSP Clinical Hospital, Faculty of Medicine, University of São Paulo, São Paulo, São Paulo 05403-900, Brazil; ⊥ Department of Biophysics, 28105 Federal University of São Paulo, São Paulo, São Paulo 04021-001, Brazil; # Sao Leopoldo Mandic Faculty, São Leopoldo Mandic Research Institute, Campinas, São Paulo 01332-000, Brazil; ∇ Faculty of Chemistry, 56724 University of the Republic, Montevideo 11800, Uruguay

**Keywords:** microneedle arrays, polypyrrole, electrochemical biosensor, interferon-alpha, interstitial fluid, point-of-care diagnostics, cytokine detection

## Abstract

Monitoring cytokines, such as interferon-alpha (IFN-α), is essential for assessing immune responses during viral infections and in immunotherapies. Here, we report the development of a microneedle-based electrochemical biosensor for detecting IFN-α in interstitial fluid (ISF), which combines minimally invasive sampling with label-free, offline analysis. The device comprises polycaprolactone (PCL) microneedles fabricated via 3D-printed molds and thermocompression, coated with polypyrrole (PPy) to enable conductivity and biomolecule immobilization. A range of PPy concentrations (10–200 mmol·L^–1^) was evaluated to optimize performance. Structural and physicochemical analyses revealed that intermediate PPy content (50 mmol·L^–1^) ensured optimal surface roughness, porosity (29.7%), and electroactive area, while preserving mechanical integrity and biocompatibility. Electrochemical impedance spectroscopy and cyclic voltammetry demonstrated a sensitive and specific response to IFN-α, with a limit of detection of 8.6 pg/mL and a linear range of up to 1000 pg/mL. Selectivity studies in gelatin matrices and cytotoxicity assays confirmed the robust performance and safety of the system. The biosensor operates via skin insertion, immunocapture, and analyte quantification after removal, enabling offline detection without complex instrumentation. These results demonstrate the potential of PPy-coated microneedles as cost-effective, scalable, and minimally invasive platforms for cytokine monitoring in point-of-care diagnostics.

## Graphical abstract

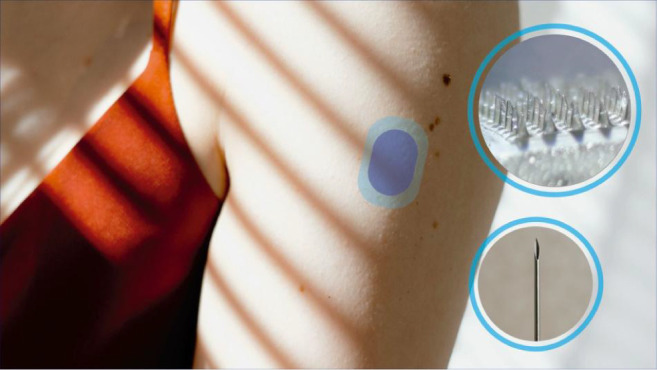

## 1 Introduction

Microneedles (MNs) represent a rapidly evolving class of biomedical devices designed to penetrate the stratum corneum, the outermost layer of the skin, without stimulating underlying nerves or blood vessels, thereby enabling minimally invasive and painless access to the dermal interstitial space.^1,2^ These miniature needles, typically ranging from tens to hundreds of micrometers in length, offer numerous advantages over traditional hypodermic needles, including reduced pain, enhanced patient compliance, and the possibility of self-administration, which is particularly beneficial for individuals requiring frequent monitoring or therapy.^3^ Over the past decade, MNs have transitioned from primarily serving as drug delivery systems to multifunctional platforms capable of sampling biological fluids and enabling biosensing applications.^4^

Among the biological fluids accessible via MNs, interstitial fluid (ISF) has gained prominence due to its proximity to systemic circulation and its composition, which mirrors the plasma profile for many biomarkers of physiological and pathological relevance.^5–7^ ISF contains critical analytes, including glucose, lactate, electrolytes, hormones, and cytokines, making it a valuable medium for real-time, noninvasive health monitoring.^8^ The development of MN-based biosensors has thus opened new avenues in point-of-care diagnostics, enabling continuous or on-demand analysis of health indicators without the logistical and procedural burdens associated with conventional assays, such as ELISA or PCR.^9,10^

A central challenge in advancing MN-based biosensors is developing materials and transduction mechanisms that simultaneously provide mechanical integrity, biocompatibility, and electrical conductivity. In this context, conductive polymers (CPs) have emerged as ideal candidates for enhancing the functional capabilities of MN systems.^11–13^ CPs such as polypyrrole (PPy) and poly­(3,4-ethylenedioxythiophene):poly­(styrenesulfonate) (PEDOT:PSS), combine the structural flexibility of polymers with the charge transport properties of semiconductors, enabling the design of conformable, stretchable, and electroactive devices suitable for biomedical integration.^14–18^ These materials support multiple modalities, including electrochemical sensing, electrical stimulation, and controlled drug release, while maintaining compatibility with soft biological tissues.^16,18,19^

Recent advances in MN-based biosensors have explored integrating conductive polymers to enhance electrochemical performance, improve charge transport, and enable real-time signal transduction. Keirouz et al.^20^ demonstrated that microneedles coated with PEDOT:PSS exhibited improved biocompatibility and conductivity, enabling their application in minimally invasive electrochemical biosensors for skin-interfacing diagnostics. Similarly, GhavamiNejad et al.^21^ integrated PEDOT:PSS within a hydrogel-based MN array for simultaneous biomarker capture and signal amplification, revealing the potential of conductive polymers in dual-function devices that merge sensing and therapeutic capabilities. Furthermore, early works by Mansoor et al.^22^ demonstrated the feasibility of combining conductive polymers, such as PPy, with microneedle structures, supporting the concept of creating electroactive interfaces suitable for flexible electronics and biosensors. More recently, Yang and coworkers proposed the development of conductive MN-based drug delivery patches using PPy, demonstrating controlled, electrically triggered release in both transdermal and inflamed skin conditions.^17,23^

Although microneedle-based sensors have advanced significantly for monitoring small molecules, such as glucose and lactate, extending this technology to the selective detection of macromolecular biomarkers, particularly cytokines, remains in its early stages.^24–26^ Recent studies have demonstrated the feasibility of integrating conductive polymers, such as PPy and PEDOT:PSS, into MN platforms to enhance electrochemical transduction and functionalization capacity for biosensing applications.^20,27^ However, translating these materials into clinically viable cytokine biosensors remains a significant challenge. This is largely due to the need for precise control over physical and chemical parameters such as porosity, surface charge, mechanical strength, and biomolecule immobilization. Furthermore, the complex interplay among electrochemical performance, biocompatibility, and functionalization efficiency in conductive polymer-coated MNs requires deeper investigation.^12,14^ In this context, the present study investigates the fabrication, multiscale characterization, and electrochemical evaluation of PPy-coated polycaprolactone (PCL) microneedles, with the aim of elucidating structure–property–performance relationships relevant to immune biosensing applications.

Among cytokines, interferon-alpha (IFN-α) stands out as a clinically and immunologically relevant biomarker. As a type I interferon, IFN-α orchestrates critical antiviral responses by activating interferon-stimulated genes and modulating innate and adaptive immunity, including the activity of dendritic cells, NK cells, and cytotoxic T lymphocytes.^28,29^ Elevated IFN-α levels are associated with acute viral infections (e.g., hepatitis, HIV, SARS-CoV-2), inflammatory responses, and autoimmune pathologies such as systemic lupus erythematosus, Sjögren’s syndrome, and dermatomyositis.^29^ Furthermore, type I interferonopathies—a group of rare, inherited autoinflammatory diseases—are also linked to persistent overactivation of the IFN pathway due to genetic mutations. These disorders often manifest with distinctive pediatric signs, such as juvenile arthritis resistant to treatment, necrotizing vasculitis, and noninfectious interstitial lung disease.^30,31^ Understanding the molecular basis and immune dysregulation associated with IFN-α in these contexts is essential for early diagnosis and the development of targeted therapies.

Moreover, exogenous IFN-α remains in clinical use for treating chronic hepatitis C, malignant melanoma, and certain hematologic cancers, despite being linked to neuropsychiatric side effects and inflammatory complications.^32,33^ These effects reinforce the importance of sensitive and real-time IFN-α monitoring in therapeutic settings. However, sensitive, conventional assays such as ELISA involve multiple incubation, washing, and amplification steps, which limit their applicability for rapid diagnostics and point-of-care use.^34^

Given its central role in antiviral defense, autoimmunity, and immunotherapy, IFN-α was selected in this study as a biologically relevant model cytokine to validate the proposed biosensing platform. The clinical importance of IFN-α, coupled with the limitations of conventional detection methods—such as their labor-intensive and time-consuming nature—underscores the need for rapid, minimally invasive, and label-free alternatives. Using IFN-α as a target enables the functional assessment of the microneedle sensor under conditions that simulate physiologically meaningful biomolecular interactions, offering a representative framework for future adaptation to a broader range of biomarkers.

The present study proposes the development and characterization of a microneedle-based electrochemical biosensor for detecting IFN-α. The system integrates PCL MNs, chosen for their biodegradability, mechanical robustness, and ease of fabrication, with a PPy coating that serves both as a conductive interface and a functional platform for biomolecule immobilization. The biosensor operates in three stages: (i) Application, where MNs penetrate the skin and contact ISF; (ii) Immuno-capture, where IFN-α selectively binds to the surface of the functionalized PPy coating; and (iii) Extraction and readout, where the MNs are removed, and the bound analyte is quantified via electrochemical impedance spectroscopy (EIS) and cyclic voltammetry (CV), enabling label-free detection through changes in charge transfer resistance and redox behavior, as shown in Figure 1.

**Figure 1:**
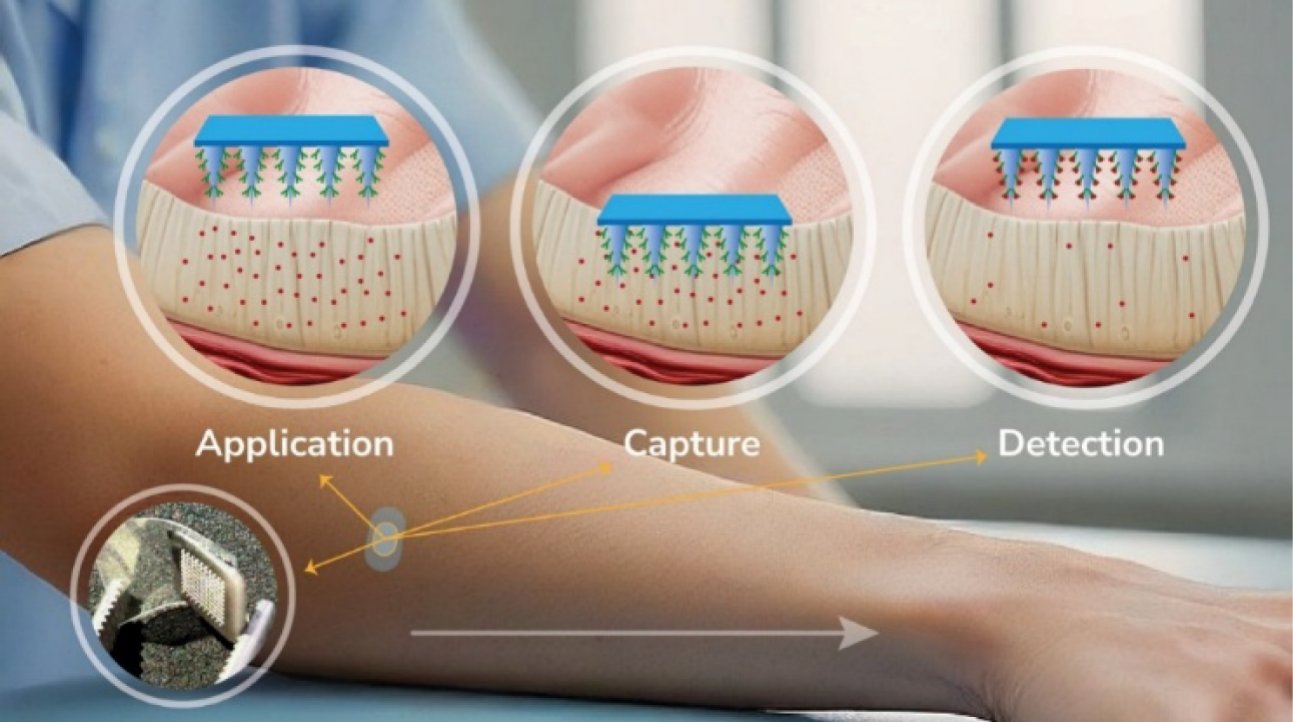
Schematic representation of the microneedle-assisted electrochemical biosensing process. The system operates in three sequential steps: (i) Application, where the microneedles penetrate the skin and establish contact with the ISF; (ii) Capture, where target biomolecules selectively bind to the functionalized microneedle surface; and (iii) Detection, where the microneedles are removed for subsequent offline electrochemical analysis.

In contrast to wearable continuous monitoring systems, the extraction and offline modality adopted here offer distinct advantages in terms of analytical reliability, system robustness, and long-term stability. As highlighted in recent literature,^35–39^ this format decouples the sensing interface from the readout electronics, thereby mitigating potential artifacts from mechanical stress, reducing power and data transmission requirements, and enabling sample preconcentration and matrix removal before analysis. This approach also enhances compatibility with standard benchtop potentiostats and portable readers, enabling cost-effective deployment in clinical and remote settings without complex integration. Additionally, the study includes a comprehensive set of morphological, structural, spectroscopic, and electronic characterizations to elucidate the interactions between PCL and PPy and to optimize the stability and performance of the biosensor. Biocompatibility assays were also conducted to ensure the safety of the system for potential clinical applications. By integrating a biodegradable polymeric matrix with a conductive coating, this work demonstrates a minimally invasive, real-time cytokine-detection platform with potential applications in personalized medicine, immunotherapy monitoring, and the diagnosis of infectious diseases.

## 2 Materials and Methods

### 2.1 Materials

Polycaprolactone (PCL, MW 70,000–90,000 g·mol^–1^, Sigma-Aldrich, cat. 440744) served as the base polymer for microneedle fabrication. Polydimethylsiloxane (PDMS) molds with pyramidal cavities (700 μm height) were provided by Micropoint Technologies Pte Ltd. Pyrrole (98%, Sigma-Aldrich, cat. 131709) was polymerized using ferric chloride hexahydrate (FeCl_3_·6H_2_O) as the oxidant. Flexible indium tin oxide (ITO)-coated polyethylene terephthalate (PET) substrates (Sigma-Aldrich, cat. 639303) featured a 60 Ω sq^–1^ surface resistivity, 1300 Å ITO thickness, and >78% transmittance at 550 nm. Microneedle arrays were affixed to the ITO/PET films using double-sided conductive carbon tape.

The IFN-α antibody and antigen used in the immunosensing assays were obtained from PBL Assay Science (USA) as part of the VeriKine Human Interferon Alpha Multi-Subtype Serum ELISA Kit (catalog no. 41110-1, lot no. 7523). Porcine gelatin was purchased from Merck (product G1890, CAS 232-554-6). The Strat-M® membrane, used as a synthetic transdermal diffusion model (25 mm diameter), was also obtained from Merck (product code SKBM02560).

### 2.2 Fabrication of PCL Microneedles

Microneedles were fabricated using a two-step thermal casting protocol based on a positive master mold generated by stereolithography (SLA) 3D printing. The master mold, featuring an array of pyramidal microneedles (700 μm in height), was designed in CAD software and printed using a Formlabs stereolithography printer (Formlabs Form 3, Formlabs Inc., USA) with high-resolution photopolymer resin. The printed mold was postcured under UV light and used to cast flexible polydimethylsiloxane (PDMS) negative molds via standard cross-linking and curing procedures.

To produce the microneedle arrays, a thermal pressing process was applied. A single PCL pellet was initially deposited into each cavity of the PDMS mold and heated at 130 °C for 50 min in a glass Petri dish to soften the polymer. The mold was then centrifuged at 5300 rpm and 40 °C for 10 min to ensure complete filling of the microneedle cavities. A second PCL pellet was subsequently added to each mold, followed by an additional thermal step at 130 °C for 10–15 min. The molds were removed and subjected to a controlled compression step, during which a 60 g weight was placed over the mold to promote flat base formation and polymer adhesion.

After drying at room temperature for 20 min, the solidified microneedles were carefully demolded and inspected under an optical microscope for uniformity and structural integrity. This process ensured consistent fabrication of high-aspect-ratio microneedles suitable for mechanical insertion and electrochemical coating.

### 2.3 Polypyrrole Polymerization on PCL Microneedles

Polymerization of pyrrole monomer on PCL microneedles was performed via oxidative polymerization.^40,41^ A medium reaction was prepared using a 1:1 (v/v) ethanol/deionized water solution (50 mL), into which the pyrrole monomer was introduced at predefined concentrations calculated using the equation *C*_py_ × *pM*_py_ × 1/*d*_py_ = *V*_py_, where *C*_py_ represents the pyrrole concentration (mol/L), *pM*_py_ is the molecular weight of pyrrole (g/mol), *d*_py_ is the pyrrole density (mL/g), and *V*_py_ is the pyrrole volume in mL per liter of solution. The solution was stirred magnetically for 1 h to initiate the prepolymerization process. Subsequently, 30 microneedle arrays were immersed in the pyrrole solution and stirred for 3 h to ensure uniform exposure to the monomer.

In parallel, a 0.25 mol L^–1^ FeCl_3_ solution was prepared and refrigerated for 1 h to control the exothermic nature of the reaction. The microneedles were then carefully transferred from the pyrrole solution into the chilled FeCl_3_ solution and left under constant agitation for 24 h to complete the polymerization of polypyrrole onto the PCL microneedle surfaces. Upon completion of the polymerization process, the microneedles underwent a rigorous washing protocol to remove unreacted species. This protocol involved sequential washing steps: a 1-h wash in ethanol/deionized water (1:1) under agitation, a deionized water wash in an ultrasonic bath for 30 min, and a final deionized water wash under agitation for 24 h.

After the washing steps, the microneedles were either vacuum-dried for 50 min or air-dried at room temperature for 4 h to ensure complete removal of residual moisture. The final polypyrrole-coated microneedles were visually inspected for uniformity and structural integrity before being used in further experiments.

### 2.4 Preparation of Indium Tin Oxide (ITO) Conductive Films

Flexible ITO-coated PET films were cut into 1.0 × 1.5 cm pieces, and the protective polymer layer covering the conductive surface was carefully removed to expose the ITO layer. To ensure proper surface cleanliness and reproducibility in subsequent electrochemical measurements, the films were subjected to a standardized three-step cleaning protocol, repeated three consecutive times. Each cycle consisted of 10 min of sequential agitation in acetone, 10 min in isopropyl alcohol, and 10 min in deionized water. After the final wash, the ITO/PET films were dried on lint-free absorbent material and stored in a clean, dust-free environment until use.

### 2.5 Electrochemical Immunosensor Fabrication

The electrochemical immunosensors were constructed using a three-electrode system comprising an ITO/PET working electrode modified with PCL microneedles coated with PPy at different concentrations, an Ag/AgCl (3 mol L^–1^ KCl) reference electrode, and a platinum wire counter electrode. The microneedles were securely attached to the cleaned ITO/PET films using conductive carbon tape to ensure stable electrical contact.

For functionalization, a 6 μL drop of anti-human IFN-α antibody solution (diluted 1:100) was applied to the working electrodes and incubated at room temperature for 60 min to allow for efficient antibody immobilization. Once dry, the modified electrodes were incubated with antigen solutions ranging from 10 to 1000 pg mL^–1^ for 5 to 60 min. Between each step, the electrodes were washed with phosphate buffer under agitation for 10 s to remove unbound material and prevent nonspecific adsorption.

Following the final washing step, the immunosensors were stored under controlled conditions for subsequent electrochemical analysis. Figure S1 shows a photograph of the microneedle integrated onto the ITO/PET conductive film in the final configuration.

### 2.6 Epidermis Mimicking and Electrochemical Testing

To simulate epidermal conditions, a 10% porcine gelatin matrix was prepared following the method described in literature.^42^ Two formulations were used: a pure gelatin sample as a control and a gelatin sample enriched with human IFN-α standard at a concentration of 1000 pg·mL^–1^. The polypyrrole-coated microneedle (PCL/PPy) sensors at 50 mmol·L^–1^, with and without prior antibody sensitization, were applied to the gelatin for immunocapture. After the interaction period, the sensors were carefully washed with phosphate buffer under agitation for 20 s to remove unbound material, ensuring specificity before electrochemical testing.

### 2.7 Material Characterization

X-ray diffraction (XRD) was performed on a Rigaku Ultima IV diffractometer using CuKα radiation in a Bragg–Brentano configuration, spanning a 2θ range of 4.0–32.0°, with a step size of 0.02° and an integration time of 10 s per step. Fourier-transform infrared (FTIR) spectra were acquired using a Shimadzu IRSpirit FTIR-ATR spectrometer. Raman spectroscopy measurements were conducted using a WITec Alpha 300-RA system equipped with a 532 nm excitation laser at ∼10 mW. Additional Raman spectra were collected using an Invia Renishaw spectrometer, coupled to a microscope with a 50× objective lens and a 785 nm diode laser.

Impedance spectroscopy for the microneedles in the solid state was performed using a Gamry Reference 3000 analyzer, with an applied AC signal of 50 mV over a frequency range of 1 Hz to 1 MHz. Atomic force microscopy (AFM) was performed using a Cypher system (Asylum Research, Inc.). The measurements were obtained in tapping mode using a silicon tip (force constant 2.6 N m^–1^, resonant frequency ∼300 kHz). Images were collected at 512 × 512 pixels across areas of 0.5–2.0 μm^2^. Topography and phase data were processed and analyzed using Gwyddion and Asylum Research software.^43^

Confocal Laser Scanning Microscopy (CLSM) images were obtained using a Zeiss LSM 710 microscope. Samples were excited at 405 nm, and emission was collected between 406 and 499 nm. Before CLSM analysis, microneedle devices were incubated with 15 μL of fluorescamine solution (0.5 mg·mL^–1^ in acetone) for 1 min to fluorescently label proteins (antibody and antigen) bound to the PPy-coated surface. After labeling, samples were rinsed to remove excess reagent and immediately imaged. Fluorescence distribution along the microneedle shafts and tips was quantified by calculating the mean fluorescence intensity using ImageJ (NIH, USA).^44^

The porosity of the MN samples was evaluated using the gravimetric method, as previously described by Wan et al.,^45^ with absolute ethanol employed as the wetting liquid to permeate the porous matrix. Briefly, dry samples were first weighed (ω_dry_) using an analytical balance. Subsequently, the microneedles were immersed in ethanol for 24 h to ensure complete pore saturation. After excess surface ethanol was gently removed using blotting paper, the wet weight (ω_wet_) was measured. The porosity (ε) was calculated according to eq 1:

1 $$\varepsilon =\frac{\omega_{\mathrm{wet}}-\omega_{\mathrm{dry}}}{\rho_{\mathrm{ethanol}}.V}\times 100$$

where ω_wet_ is the weight of the ethanol-saturated sample (g), ω_dry_ is the dry weight of the sample (g), ρ_ethanol_ is the density of absolute ethanol (0.789 g·cm^–3^ at 25 °C), and *V* is the geometric volume of the sample (cm^3^), calculated from the microneedle patch dimensions. The polymer density (PCL) was considered to be 1.145 g·cm^–3^. All measurements were performed in triplicate.

Specific surface area and total pore volume were determined using nitrogen adsorption–desorption measurements performed with a Micromeritics ASAP 2010 instrument. Before analysis, samples were degassed under vacuum at 80 °C for 12 h. The Brunauer–Emmett–Teller (BET) method was applied to determine surface area from adsorption isotherms in the relative pressure range of 0.05–0.30. The total pore volume was obtained from the amount of nitrogen adsorbed at a relative pressure close to unity (P/P_0_ ≈ 0.99), while the average pore diameter was estimated using the Barrett–Joyner–Halenda (BJH) method from the desorption branch of the isotherms.

Thermogravimetric analysis (TGA) was performed using a TGA Q500 V20.13 Build 39 instrument (Module: TGA; Serial No: 0500-1572). Microneedles with five different PPy concentrations and a control sample of PCL without PPy were analyzed. Each microneedle sample was cut into four similar pieces to meet the maximum weight requirements of the equipment, with a quarter of a microneedle placed on the scale for thermogravimetric measurements. The samples were heated from room temperature to 800 °C at 10 °C/min under a nitrogen atmosphere to evaluate thermal degradation profiles and estimate residual polypyrrole content. The thermograms were analyzed to compare thermal stability, decomposition steps, and the influence of PPy concentration on the thermal behavior of the composite microneedles.

The mechanical performance of microneedles was further evaluated through failure force analysis.^46,47^ Each MN array was positioned base-down on the testing stage of an Anton Paar MCR 502 rheometer, equipped with a 25 mm parallel plate. The top plate was lowered vertically at a constant rate of 0.035 mm/s until the microneedles fractured. The force–displacement data were recorded, and the failure force was defined as the peak force observed before structural failure. The force–displacement curves were analyzed using a four-parameter logistic function to describe the failure behavior of the microneedles, as shown in eq 2:

2 $$F\left(x\right)=\frac{a}{1+e^{-c(x-d)}}+b$$

where *F­(x)* is the applied force as a function of displacement *x*, *a* represents the amplitude of the force increase, *b* is the baseline force, *c* is the growth rate, and *d* is the displacement value at the inflection point. This fitting model enabled the estimation of the mechanical response range and provided insight into the stiffness and failure threshold of the MN arrays under compressive loading.

In addition to the mechanical tests, optical coherence tomography (OCT) was used to visualize the depth of microneedle insertion into Parafilm® layers.^47^ The cross-sectional images were acquired using a Callisto OCT imaging system (Thorlabs, USA) with a wavelength of 930 nm. The insertion profiles were analyzed using ImageJ (NIH, USA) to determine the depth of penetration. For each condition, three independent replicates were performed, and one hundred microneedles (*N* = 100) were analyzed per replicate to ensure statistical robustness. These quantitative data complemented the rheological force measurements, enabling a comprehensive assessment of insertion efficiency and mechanical performance.

### 2.8 In vitro Cytotoxicity Evaluation of PCL/PPy Microneedles

To evaluate the biocompatibility of the MN patches composed of PCL and PPy, an in vitro cytotoxicity assay was performed using HaCaT keratinocytes and 3T3 fibroblasts. Both cell lines were cultured in complete Dulbecco’s Modified Eagle Medium and seeded in 96-well plates at a density of 1 × 10^4^ cells per well. After 24 h of incubation at 37 °C in a 5% CO_2_ atmosphere, PCL and PCL/PPy MNs (cut into uniform fragments) were sterilized by UV irradiation and subsequently added to the wells in direct contact with the cells. After 24 h of exposure, the medium was replaced with MTT solution (0.5 mg/mL in DMEM), and the cells were incubated for 4 h under the same conditions. The resulting formazan crystals were solubilized with 200 μL of DMSO, and the absorbance was measured at 570 nm using a microplate reader (SPARK 10M, TECAN). Cell viability was calculated relative to untreated control cells using the following equation:

3 $$\mathrm{cell}\;\mathrm{viability}=\frac{\left(Abs_{570}-{Abs}_{630}\right)\mathrm{treated}\;\mathrm{cells}}{\left(Abs_{570}-{Abs}_{630}\right)\mathrm{untreated}\;\mathrm{cells}}$$

### 2.9 Electrochemical Characterization

The electrochemical performance of the immunosensors was evaluated using a Metrohm Autolab PGSTAT 302N system equipped with an FRA2 impedance module, and the data were analyzed with NOVA 2.1.3 software. All measurements were conducted in a 0.1 mol L^–1^ KCl solution containing 5 mmol L^–1^ [Fe­(CN)_6_]^3–/4–^ as the redox probe. Electrochemical impedance spectroscopy (EIS) was performed at the half-wave potential (approximately 240 mV), as determined by cyclic voltammetry, over a frequency range of 0.1 Hz to 30 kHz.

## 3 Results and Discussion

### 3.1 Morphological and Porosity Modulation by PPy Coating

PCL microneedles were produced using a positive mold fabricated via 3D printing, followed by PDMS replica molding to generate negative molds.^47^ The overall fabrication and functionalization workflow is summarized schematically in Figure 2A, which illustrates the sequence of PCL microneedle formation, in situ pyrrole polymerization, antibody immobilization, and the subsequent offline electrochemical detection process. The final microneedle arrays exhibited a well-defined pyramidal geometry, as observed in the representative SEM images (Figure 2B).

**Figure 2:**
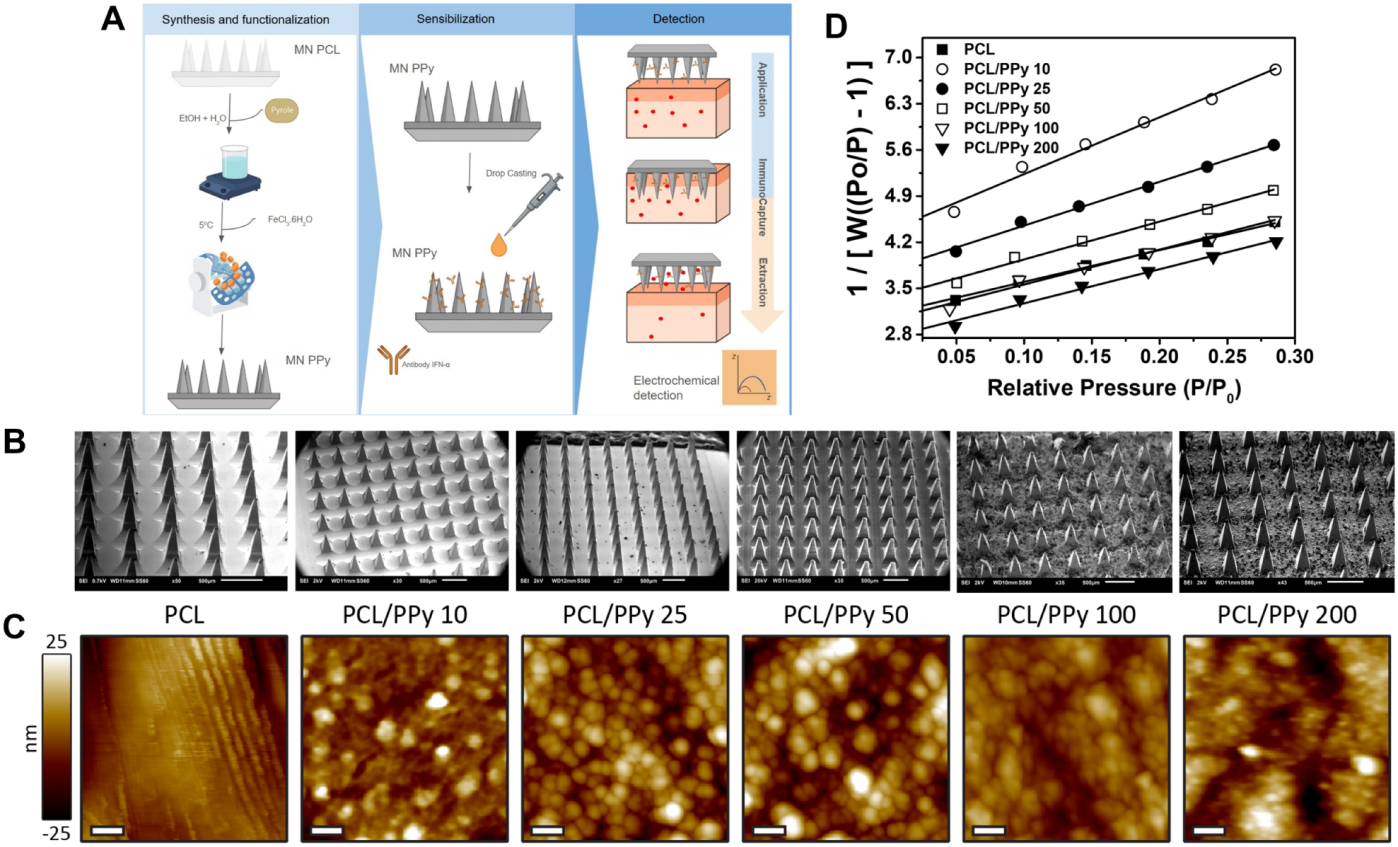
(A) Schematic illustration of the fabrication and sensing workflow: in situ pyrrole polymerization on PCL microneedles, antibody immobilization by drop casting, and the offline electrochemical detection sequence (application → immunocapture → extraction → readout). (B) SEM images of PCL and PCL/PPy microneedles prepared at different PPy concentrations, showing progressive surface roughening upon polymer deposition. (C) AFM topographic images and corresponding line profiles of the same samples, evidencing nanoscale changes in texture and roughness. The false-color vertical scale applies to all images; lateral scale bars = 100 nm. (D) BET plots from nitrogen adsorption for PCL and PCL/PPy microneedles, with specific surface area estimated from the linear region of the BET equation (P/P_0_ = 0.05–0.30); the increased slope reflects the enlargement of accessible surface area after PPy incorporation.

To enhance electrochemical activity and improve surface properties, the microneedles were functionalized with PPy via in situ oxidative polymerization. This was achieved by immersing the PCL microneedle arrays in aqueous pyrrole solutions of varying concentrations (10–200 mmol·L^–1^), followed by exposure to FeCl_3_·6H_2_O as an oxidizing agent. The general reaction involved in the polymerization is shown below.^40,41^

$$C_{4}H_{5}\mathrm{N + 2}{\mathrm{.3FeCl}}_{3}\cdot {\mathrm{6H}}_{2}O\to 0{\mathrm{.25C}}_{4}H_{3}N_{4}^{+}{\mathrm{Cl}}^{-}\mathrm{+ 2HCl + 2.}{\mathrm{3FeCl}}_{2}\cdot {\mathrm{6H}}_{2}O$$

This process led to the deposition of a conformal PPy layer on the microneedle surface, with distinct morphological changes evident in the SEM images taken after polymerization (Figure 2B). The resulting PPy coatings enhanced microneedle roughness and introduced nodular features whose characteristics varied with PPy concentration (Figure 2B,C). This morphological evolution was further investigated using AFM and nitrogen physisorption analyses to quantify changes in nanoscale organization and surface area.

AFM provided detailed insights into the surface topography of PCL microneedles functionalized with increasing concentrations of PPy. As shown in Figure 2C, pristine PCL surfaces exhibited a relatively smooth, ordered pattern with fibrillar features aligned with the mold replication direction. These striations are not intrinsic to the PCL itself but reflect the negative replica of the silicone mold used during thermal casting.

Upon the incorporation of 10 mmol·L^–1^ of PPy, the microneedle surface became notably rougher, with the emergence of nodular nanostructures indicative of early PPy phase separation within the polymer matrix. These nodules are uniformly distributed, suggesting partial miscibility between PCL and the PPy phase at low concentrations.

With increasing PPy concentrations from 25 to 50 mmol·L^–1^, the nodular domains became denser and more defined, revealing a progressive aggregation of PPy clusters on the surface. This observation supports the formation of localized PPy-rich regions, likely driven by phase segregation mechanisms. At 100 mol·L^–1^, surface roughness continued to increase, and the nodules began to coalesce into broader domains, creating heterogeneous surface features.

At the highest tested concentration (200 mmol·L^–1^), the microneedles displayed a highly irregular and coarse morphology, characterized by large, poorly connected domains and heterogeneous topography. These discontinuities at the surface are consistent with overaggregation of PPy and poor interfacial compatibility with the PCL matrix, which may compromise mechanical homogeneity and sensor performance.

Quantitative analysis of surface roughness based on the root-mean-square deviation (RMSD) confirmed the trend observed qualitatively in AFM images. As illustrated in Figure S2, RMSD values increased sharply with PPy concentration, particularly between 10 and 50 mmol·L^–1^, after which a plateau-like behavior was observed. This suggests that roughness saturates at higher PPy content, possibly due to a limit in surface coverage or saturation of phase-separated domains.

Nitrogen adsorption–desorption isotherms were obtained to evaluate the porous structure and surface characteristics of PCL and PCL/PPy microneedles (Figure S3). All samples exhibit type IV­(a) isotherms with H3 hysteresis loops according to the IUPAC classification, indicating the presence of mesopores with slit-like geometries formed by the aggregation of plate-like domains.^48^ The absence of a plateau at high relative pressures (P/P_0_ > 0.9) further supports the presence of interparticle voids and open mesostructures, consistent with nonrigid aggregates.^48^

The BET plots (Figure 2D) show good linearity over P/P_0_ = 0.05–0.30, validating the model’s applicability. Surface area, pore volume, and pore width were extracted from BET and BJH analyses and are summarized in Table 1. An initial reduction in surface area is observed at low PPy concentration (10 mmol·L^–1^), from 439 m^2^·g^–1^ (PCL) to 268 m^2^·g^–1^. This drop is attributed to pore-blocking or coating effects arising from the conformal deposition of PPy on the PCL surface. However, with further PPy incorporation (≥25 mmol·L^–1^), the surface area progressively increases, reaching values comparable or superior to pure PCL at 200 mmol·L^–1^ (439.4 m^2^·g^–1^). The pore volume follows a similar increasing trend, while the mean pore width remains nearly constant (∼32.5 Å) across all samples, indicating that PPy increases pore density rather than size.

**Table 1:** Surface Area, Pore Volume, and Average Pore Diameter for Pristine PCL and PPy-Coated Microneedle Samples Prepared with Increasing Pyrrole Concentrations^a^ ^b^ ^c^

**Microneedles**	**Surface area (m** ^**2**^ **/g)**	**Pore volume (cm** ^**3**^ **/g)**	**Pore width (Å)**	**Porosity (%)**
PCL	439.133	0.345	32.944	14.05 ± 2.78
PCL/PPy 10	268.037	0.229	32.716	11.87 ± 2.74
PCL/PPy 25	333.783	0.277	32.468	13.49 ± 3.17
PCL/PPy 50	382.440	0.319	32.426	29.73 ± 5.60
PCL/PPy 100	418.386	0.348	32.530	16.70 ± 4.94
PCL/PPy 200	439.434	0.374	32.610	13.89 ± 2.72

a Surface area and pore parameters were determined by nitrogen adsorption–desorption measurements using the BET and BJH models.

b Porosity values were calculated gravimetrically and reflect the bulk open volume fraction.

c Error bars represent standard deviations based on triplicate measurements.

Interestingly, the bulk porosity (%) shows a nonmonotonic trend, with a marked peak at 50 mmol·L^–1^ (29.7 ± 5.6%). This suggests the formation of additional macro- or mesopores not fully captured by N_2_ physisorption, likely due to partial phase separation or heterogeneous PPy distribution within the PCL matrix.

This evolution in porosity aligns with the chemical and structural reorganization induced by the incorporation of PPy into the PCL matrix.^49,50^ At intermediate concentrations (25–50 mmol·L^–1^), the porous framework becomes more accessible, potentially due to partial phase segregation and pore-forming mechanisms driven by the self-assembly of PPy domains during polymerization. At higher concentrations (≥100 mmol·L^–1^), the porosity reaches a plateau, suggesting the saturation of accessible pore sites or densification of the polymer–PPy interface. These features observed in the textural analysis complement the surface-morphology findings discussed earlier, supporting the formation of interconnected, concentration-dependent mesostructures within the microneedle architecture.^49,51,52^

### 3.2 Mechanical Performance and Skin Insertion Capability

Mechanical tests were conducted to assess the structural robustness of the microneedles and their ability to resist forces relevant to skin insertion.^46,47^ Compression tests revealed a clear dependence of mechanical strength on PPy concentration (Figure 3). The stress–strain curves indicate that PCL/PPy microneedles exhibit increasing resistance to deformation with increasing PPy content, up to 50–100 mmol·L^–1^. This behavior suggests that incorporating PPy at intermediate concentrations enhances stiffness, possibly by promoting matrix densification and strengthening polymer chain interactions. The maximum force required to compress the microneedles increased from 23.7 ± 0.6 N in pure PCL to 47.3 ± 0.7 N and 46.9 ± 0.6 N for the 50 and 100 mmol·L^–1^ formulations, respectively.

**Figure 3:**
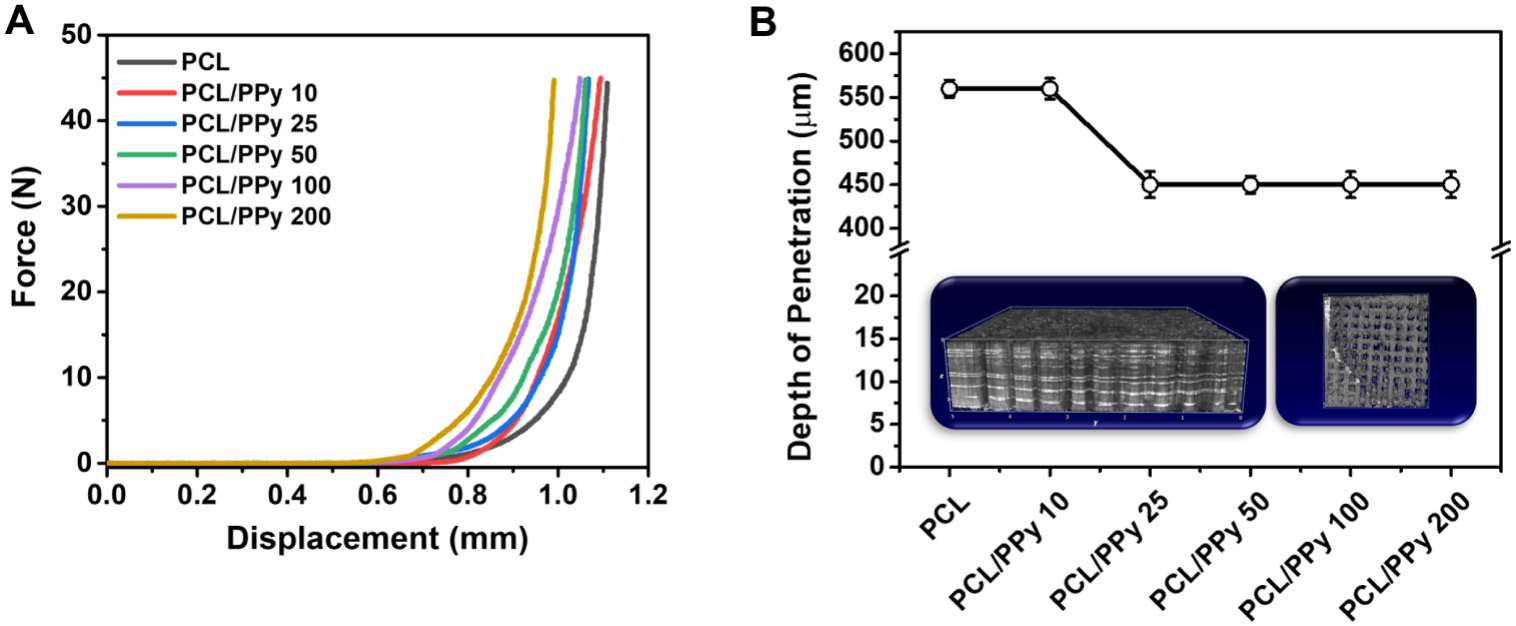
(A) Compression stress–strain curves of pristine and PPy-coated microneedles, showing that intermediate PPy concentrations (50–100 mmol·L^–1^) enhance resistance to deformation. (B) Penetration depth into Parafilm M® under a constant force of 30 N, with 3D OCT images (insert) confirming consistent intradermal access (>400 μm) across all formulations and absence of tip damage after insertion.

To quantitatively model the microneedle deformation under compression, the mechanical response curves were fitted using a four-parameter logistic function,^53^ allowing accurate extraction of the maximum force (*F*), deformation range (*d*), slope (*c*), and energy dissipation (*E*) values from the stress–strain data (Table 2).

**Table 2:** Mechanical Parameters Extracted from Compression Tests of Pristine and PPy-Coated PCL Microneedles, Including Maximum Force (*F*), Displacement at Maximum Force (*d*), Logistic Slope (*c*), and Deformation Energy (*E*)­^a^

**Microneedles**	***F*** (N)	***d*** (mm)	***c*** (mm^**–1**^)	***E* (mJ)**
PCL	23.69 ± 0.64	1.06 ± 0.003	11.71 ± 0.04	559
PCL/PPy 10	30.71 ± 0.66	1.00 ± 0.002	18.69 ± 0.10	803
PCL/PPy 25	38.11 ± 7.70	1.09 ± 0.025	10.03 ± 0.11	441
PCL/PPy 50	47.27 ± 0.73	1.03 ± 0.002	12.63 ± 0.06	1993
PCL/PPy 100	46.87 ± 0.55	0.97 ± 0.002	13.88 ± 0.08	3080
PCL/PPy 200	39.38 ± 0.72	0.93 ± 0.002	12.88 ± 0.09	2016

a All values represent means of triplicate measurements.

This reinforcement effect is also reflected in the deformation energy, which increased more than 5-fold for the 50 mmol·L^–1^ sample (1993 mJ) and over 5-fold for the 100 mmol·L^–1^ sample (3080 mJ) compared to pure PCL (559 mJ). These results indicate that PPy at intermediate concentrations effectively contributes to the mechanical integrity of the microneedle matrix, possibly through stronger intermolecular interactions and increased network cohesion. Interestingly, at the highest PPy concentration tested (200 mmol·L^–1^), the mechanical performance slightly declined (39.4 ± 0.7 N; 2016 mJ), possibly due to PPy aggregation or uneven distribution on the microneedle surface, as inferred from AFM topography and surface area trends in BET analysis.

The displacement parameter (*d*), associated with microneedle deformation range, remained within 0.93–1.09 mm across all formulations, indicating comparable structural elasticity. The slope factor (*c*) and logistic regression analysis confirmed reproducible mechanical behavior within the working range of skin deformation.

To validate the functional performance of the microneedles, a Parafilm M® penetration test was employed under a standardized force of 30 N (Figure 3). All formulations reached depths exceeding 400 μm, which is above the thicknesses of the stratum corneum and the upper epidermis, thereby confirming their mechanical suitability for intradermal access.^47^ No structural failure or tip deformation was observed postinsertion, even in the softer PCL samples, indicating that the thermal casting and drying method used in fabrication was appropriate.

Altogether, these findings demonstrate that moderate concentrations of PPy not only enhance mechanical resistance and energy dissipation but also preserve sufficient elasticity and structural resilience, thereby ensuring reliable skin penetration.

### 3.3 Structural and Spectroscopic Characterization of PPy-Coated Microneedles

X-ray diffraction (XRD) patterns revealed key structural features of the PCL/PPy microneedles and their evolution with increasing PPy concentration. As shown in Figure 4A, the characteristic peaks at 2θ ≈ 21.3° and 23.8° correspond to the (110) and (200) crystalline planes of semicrystalline PCL, consistent with literature reports for this polymer.^54^ As PPy is progressively added, these diffraction peaks broaden and attenuate, particularly at concentrations above 50 mmol·L^–1^. This trend reflects a disruption of the crystalline domains of PCL, likely due to the incorporation of disordered PPy chains within the polymer matrix. The structural interference introduced by PPy induces amorphization and reduces long-range order, a behavior often observed in polymer–conductive filler composites.^55^

**Figure 4:**
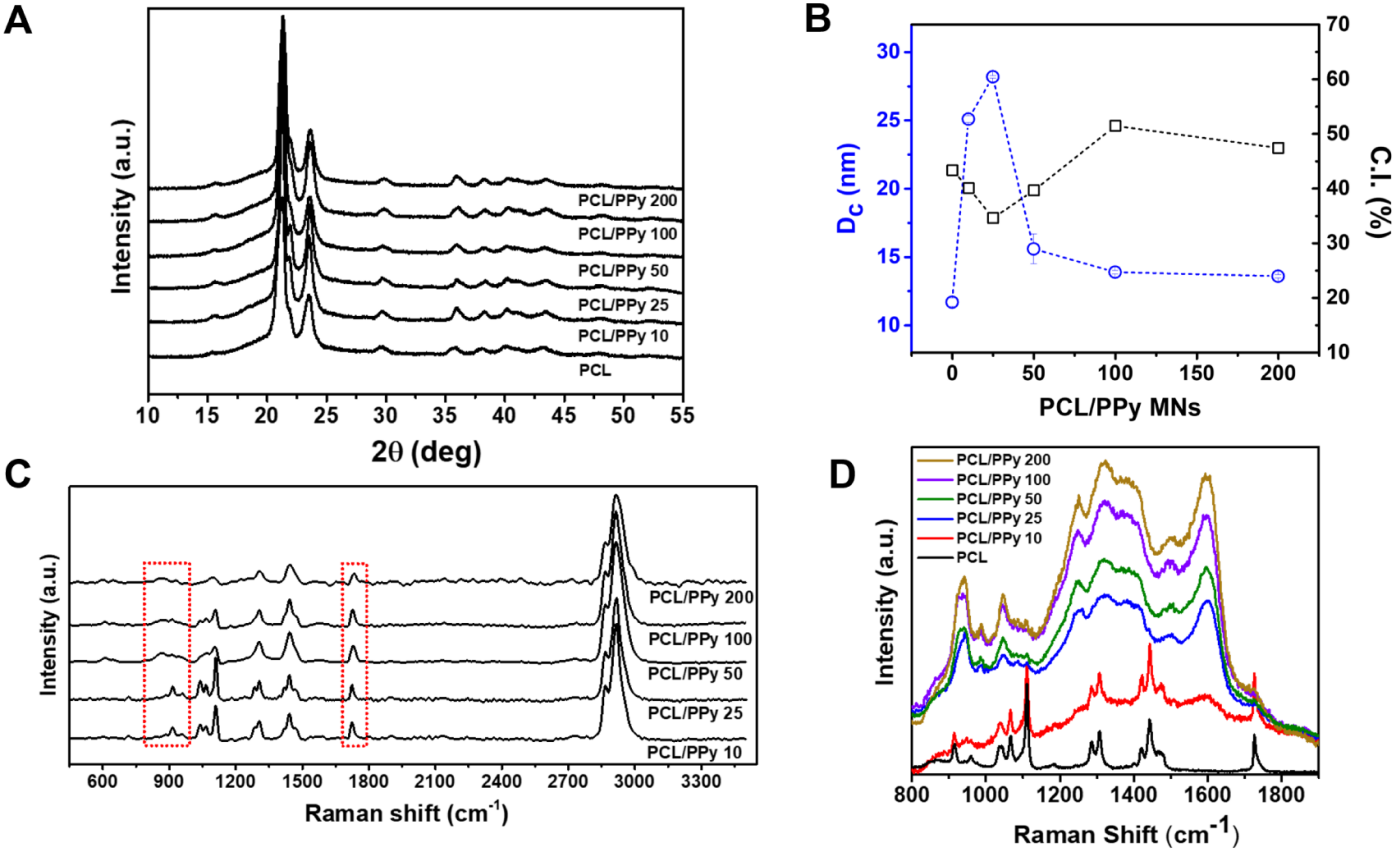
(A) XRD patterns of PCL and PCL/PPy microneedles showing the (110) and (200) crystalline reflections and their progressive broadening with increasing PPy content. (B) Crystallite size (D, blue symbols) and crystallinity index (CI%, black squares) as a function of pyrrole concentration. (C) Raman spectra acquired at 532 nm excitation and (D) 785 nm excitation, highlighting the vibrational signatures of PPy and PCL and the resonance enhancement of polaronic bands at higher PPy loadings. The red boxes in (C) indicate the main PPy vibrational modes, including C–H out-of-plane deformation (∼936 cm^–1^), C–H in-plane deformation (∼1083 cm^–1^), ring stretching (∼1237 cm^–1^), and inter-ring C=C stretching (∼1587 cm^–1^).

Moreover, Figure 4B shows a quantitative analysis of crystallite size (D_c_) and interplanar spacing (d), derived from the Scherrer equation and Bragg’s law, respectively. The crystallite size D_c_ increases up to ∼27 nm at 25 mmol·L^–1^, suggesting that low concentrations of PPy induce an enhancement of PCL crystallite mean size, possibly via nucleation effects or local confinement. However, at higher PPy concentrations, D_c_ decreases significantly to ∼13 nm, indicating that elevated PPy content introduces substantial lattice disorder and restricts crystallite development. Meanwhile, the interplanar spacing (d) remains relatively constant, suggesting that the local lattice structure of PCL is preserved despite the disruption in long-range order.

Additionally, the crystallinity index (CI%) provides further evidence of these structural changes. CI% initially drops at 10 mmol·L^–1^, reflecting the early disruption of PCL ordering and a possible increase in the amorphous-to-crystalline region ratio. As the PPy concentration increases from 25 to 100 mmol·L^–1^, the CI% rises progressively, reaching values close to 50%, before plateauing at higher concentrations (Figure 4B). This evolution suggests that moderate PPy content can promote local chain ordering and partial recrystallization, whereas higher concentrations favor a structural equilibrium with coexisting amorphous and crystalline domains.

These findings are consistent with the morphological and porosity data presented earlier, which indicate that higher PPy loadings result in greater surface heterogeneity and phase separation. Taken together, the XRD results confirm that PPy acts as a structural modulator in the PCL matrix, initially supporting partial recrystallization, and later driving amorphization and nanostructural reorganization within the microneedle composites.^56^

TGA and derivative thermogravimetry (DTG) were used to investigate the thermal stability and decomposition behavior of the PCL/PPy microneedle composites (Figure S4). Pure PCL exhibits a single, well-defined degradation event centered at 410 °C, in agreement with the typical ester pyrolysis and main-chain scission reported for this polymer.^57^ The degradation onset occurs near 360 °C, with a sharp mass loss of approximately 95%, reflecting the thermal decomposition of the polymer backbone.^58^ As PPy is incorporated into the matrix, distinct changes in the thermal profile emerge. At lower PPy concentrations (10–25 mmol·L^–1^), the thermal behavior remains similar to that of neat PCL, with only slight deviations in degradation onset and rate. However, at intermediate PPy concentrations (50–100 mmol·L^–1^), the onset of degradation shifts to lower temperatures, and the mass loss becomes more gradual. This suggests that PPy affects the integrity of the polymer network, possibly by introducing microstructural discontinuities and enhancing chain mobility. The broadening of the DTG peaks at these concentrations indicates a more complex, less cooperative degradation mechanism.

At 200 mmol·L^–1^ PPy, the TGA and DTG curves exhibit two distinct degradation steps. The first, occurring around 300 °C, corresponds to the initial decomposition of PPy-rich domains and loosely bound segments of the PCL matrix. The second event, near 390 °C, is attributed to the degradation of the remaining PCL-rich regions. This two-step profile indicates partial phase separation and the formation of thermally heterogeneous microdomains within the composite. The residual mass after heating to 600 °C also increases with PPy content, consistent with the known thermal stability and carbonaceous residue of doped polypyrrole.

The TGA/DTG data clearly show that increasing PPy content alters the degradation behavior of PCL, with higher concentrations leading to reduced thermal stability and more complex decomposition pathways. These effects reflect not only macroscopic changes in thermal stability but also deeper alterations in molecular interactions and electronic structure. To elucidate these aspects, particularly the evolution of conjugation, doping, and phase distribution within the composite, we employed Raman spectroscopy, as described below.

Raman spectroscopy was employed to investigate molecular interactions and structural evolution in PCL/PPy composites, providing insight into the conjugation states, phase distribution, and PPy doping level as a function of concentration (Figure 4C,D). The two excitation wavelengths were chosen to highlight the vibrational modes of PCL and PPy selectively. The Raman spectrum of pure PCL is dominated by characteristic bands assigned to C=O stretching (∼1722 cm^–1^), C–H bending (∼1441 and 1305 cm^–1^), and O–C–O stretching (∼1105 cm^–1^), shown in Figure 4C, in agreement with literature values for semicrystalline polyesters.^59–61^ The broadening of the PCL peaks is observed with increasing PPy concentration, and, particularly, the PCL C=O stretching mode exhibits a blueshift from ∼1722 to 1733 cm^–1^, as depicted in Figure 4C. This suggests molecular-level interactions between PCL and PPy, likely via the PCL ester group. Upon incorporation of PPy, distinct spectral features emerge in the 900–1600 cm^–1^ range. These include bands attributed to C–H out-of-plane deformation (∼936 cm^–1^), C–H in-plane deformation (∼1083 cm^–1^), ring stretching (∼1237 cm^–1^), and inter-ring C=C stretching (∼1587 cm^–1^), which are diagnostic of the PPy backbone.^62,63^ As the PPy concentration increases, the intensity of these peaks becomes more pronounced, confirming the progressive incorporation of the conductive polymer into the microneedle matrix.

The band at ∼1083 cm^–1^ (C–H in-plane deformation) exhibits an intensity increase with rising PPy content, which is commonly associated with the presence of doped structures and the formation of polarons or bipolarons along the polymer chain.^64^ Similarly, the broad band around ∼1587 cm^–1^, seen mainly with the 785 nm excitation, attributed to the C=C stretching of conjugated pyrrole rings, shifts slightly and broadens with increasing PPy levels, indicating changes in conjugation length and chain disorder. These observations suggest that higher concentrations of PPy result in higher oxidation states and partial phase segregation, consistent with the loss of crystallinity observed in the XRD results.

As shown in Figure 4C,D, the Raman spectra vary with the excitation wavelength. The 785 nm excitation enhanced PPy signals due to a resonance with the delocalized charge carriers (polarons/bipolarons),^65^ whereas the 532 nm excitation clearly reveals the features of the PCL matrix. This wavelength-dependent enhancement with the 785 nm further confirms the presence of doped electronic states in PPy and underscores the importance of spectroscopic selectivity when probing conjugated polymer composites.

The enhancement of the PPy Raman modes with the 785 nm excitation, particularly at concentrations above 100 mmol·L^–1^, suggests the formation of PPy-rich domains within the PCL matrix. This spectroscopic evidence supports the hypothesis of local phase segregation and the coexistence of conductive and insulating regions in the composite, reinforcing conclusions drawn from TGA and XRD analyses.

Taken together, the Raman analysis supports the interpretation that the incorporation of PPy modifies the local structure and electronic environment of the composite. The vibrational fingerprints reveal a transition from a more homogeneous PCL matrix to a heterogeneous, doped PPy–PCL hybrid, with tunable conjugation and molecular ordering depending on PPy concentration. These findings align with the morphological, structural, and thermal data, providing critical molecular-level evidence of the composite architecture, which is relevant to its electrical and sensing performance.

### 3.4 Electrochemical Properties in Solid-State and Solution

EIS was employed to probe the charge transport behavior and electrical response of the PCL/PPy microneedles in both solid-state and solution environments. First, we study the impedance response of microneedles in the solid state to evaluate their intrinsic electrical conduction properties. Impedance data could be obtained only for samples with higher PPy concentrations (50–200 mmol L^–1^) because of their lower impedance values (Figure S5). In all these cases, the electronic transport was modeled using the parallel combination of resistance (R) and a constant phase element (CPE), and an additional series resistance (R_s_) was only necessary for the sample with a higher PPy content, which was negligible compared to the bulk resistance in the other cases. The Nyquist plots and extracted circuit model fitting parameters (R, Q, α) reveal how PPy incorporation affects the microneedle’s electronic transport in the solid state.^66^ The Nyquist plots indicate that the electrical transport is purely electronic, considering that only the real impedance response is preserved at the lowest frequencies.^67^

The fitted resistance (R) decreases significantly with higher PPy content, ranging from 63.25 kΩ (higher PPy, i.e., 200 mmol L^–1^) to 9.70 GΩ (lower PPy, i.e., 50 mmol L^–1^), as shown in Figure S6. This suggests that, although PPy is intrinsically conductive, its distribution within the PCL matrix plays a dominant role in modulating charge-transport efficiency. The capacitance-related element (Q) decreases with increasing PPy concentration, indicating a reduction in charge storage capacity, which may be attributed to changes in porosity and interface properties. The α parameter (ranging from ∼0.8 to 0.9) indicates a nearly ideal capacitor-like behavior and increased charge-transport heterogeneity, with a more disordered conduction mechanism at higher PPy concentrations, as depicted in Figure S6.

The additional electrical series resistance (R_s_) of 5381 Ω, observed only in the sample with the higher PPy content (200 mmol L^–1^), indicates a semiconductive nature influenced by the percolation of PPy conductive domains, suggesting a transition from well-connected percolation pathways to a more heterogeneous, less conductive network.

The increased amorphization of the PCL crystalline structure with increasing PPy content observed in the XRD analysis suggests that the PPy forms a percolation structure, permitting the electronic transport across the microneedle’s hybrid structure.^68^ The Raman spectra provide additional evidence of these structural-electronic interactions. The shifts in the C=C backbone stretching (∼1590–1610 cm^–1^) and the presence of polaronic and bipolaronic species confirm modifications in the conjugation length and electronic structure of PPy.^65^ At higher PPy concentrations, these Raman peaks are enhanced in agreement with the increased ability of the electronic conducting properties of the microneedles.

These findings confirm that the charge transport behavior in PCL/PPy microneedles is intrinsically linked to the structural evolution of the composite.^69^ The structural-electrical correlation observed across XRD, Raman, and impedance spectroscopy data underscores the importance of optimizing PPy dispersion and processing strategies to balance mechanical integrity, electrochemical activity, and conductivity. Porosity measurements (Table 1) further support this correlation, revealing a nonmonotonic trend:^12^ while the initial addition of PPy reduced porosity, a pronounced increase was observed at 50 mmol·L^–1^, likely due to morphological restructuring and the formation of open conductive domains, followed by a decrease at higher concentrations, consistent with densification and phase segregation. These changes affect both charge storage capacity and ion transport, highlighting the need to fine-tune the composite architecture. This structure–property relationship also plays a critical role in determining the electrochemical performance of the microneedles in solution, as discussed below.

The electrochemical behavior of the PCL/PPy microneedles was further investigated in 0.1 mol L^–1^ KCl solution using ferricyanide as a redox probe, employing CV and EIS to assess their charge transfer characteristics (Figure S7). These solution-based electrochemical measurements provide valuable insights into electron-transfer kinetics and interfacial charge transport, enabling direct comparison with previously discussed solid-state impedance results.

The CV curves exhibit a straightforward quasi-reversible redox process for the ferricyanide system, with increasing current response as the PPy concentration increases. The peak current enhancement observed at higher PPy loadings (100–200 mmol L^–1^) indicates that PPy enhances the electrochemical activity and charge-transfer properties of the microneedles. However, at very high PPy concentrations (200 mmol L^–1^), the voltammogram exhibits a broader peak separation (ΔE_p_) (Figure S7), which is consistent with previously observed phase segregation effects in XRD and Raman data.

These trends align with the solid-state impedance results, in which higher PPy concentrations initially improved charge transport but, beyond a threshold, led to structural disorder and a loss of connectivity within the polymeric network.^69^ The observed CV behavior suggests that, in solution, ion accessibility and charge transfer efficiency are similarly affected by PPy percolation and aggregation effects.

The Nyquist plots from the EIS measurements in 0.1 mol L^–1^ KCl further confirm the charge transfer characteristics observed in CV. The data exhibit semicircular and Warburg-type behavior, characteristic of electron transfer kinetics coupled with ion diffusion at the electrode interface. As the PPy concentration increases, the charge transfer resistance (R_ct_) initially decreases, indicating that PPy incorporation enhances electrical conductivity and facilitates electron transfer at the interface. However, above 100 mmol·L^–1^, the R_ct_ values reach a plateau, suggesting that charge transport is limited by phase segregation.

These observations closely correlate with the solid-state impedance results: moderate PPy concentrations enhanced conductivity, whereas excessive loading disrupted charge percolation.^69^ The solution-based EIS measurements reinforce this conclusion, as the electrochemical charge transfer processes follow the same trend. Together, these results suggest that an optimal PPy concentration, between 50 and 100 mmol·L^–1^, maximizes charge transport without compromising structural integrity, which is crucial for the reliable operation of microneedle-based biosensing applications.

### 3.5 Biosensing Performance: Sensitivity, Specificity, and Complex Matrix Application

Based on the optimized electrochemical properties described above, we next investigated the biosensing capabilities of the MN system. The integration of the PCL/PPy MN array onto a flexible ITO/PET conductive substrate (Figure S1) enabled the development of a conformable and low-resistance electrochemical platform suitable for direct contact with biological tissues. This configuration ensures effective signal transduction even under mechanical deformation, making it particularly suitable for biomarker detection strategies.

To assess biosensing performance, the MNs were evaluated in three functional states: (i) bare PCL/PPy MNs, (ii) MNs modified with anti-human IFN-α monoclonal antibodies, and (iii) after exposure to the IFN-α antigen. Confocal laser scanning microscopy Z-stack reconstructions (Figure 5A) revealed a progressive increase in fluorescence intensity across these states, validating the successful, homogeneous immobilization of antibodies and subsequent antigen recognition across the MN surface.

**Figure 5:**
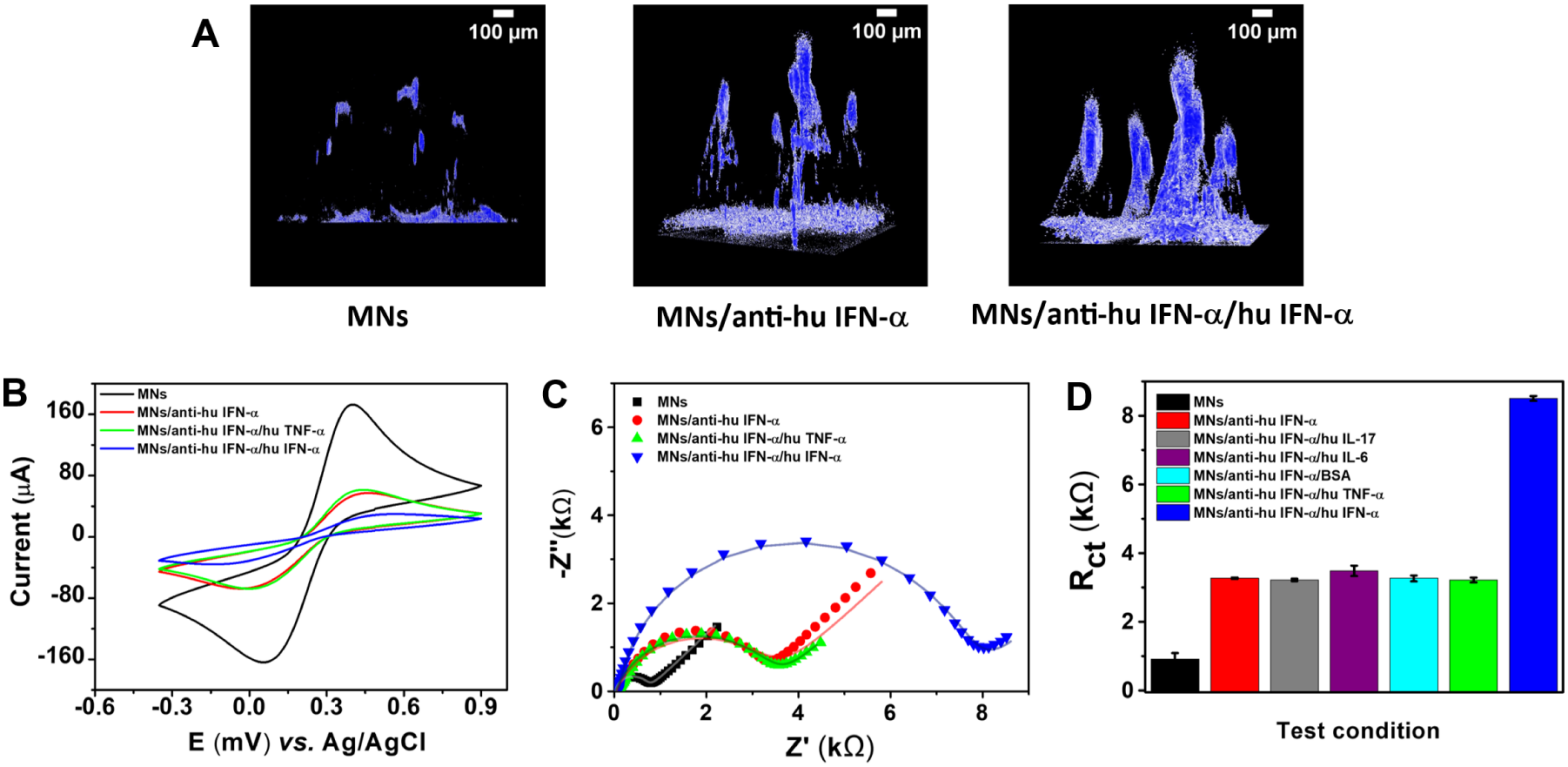
(A) CLSM images of PCL/PPy microneedle (MN) arrays at three stages of functionalization: unmodified MNs, after immobilization of anti-human IFN-α antibodies, and after incubation with human IFN-α antigen. The progressive fluorescence enhancement across the microneedle shafts and tips confirms successful and homogeneous antibody immobilization followed by specific antigen binding. (B) Cyclic voltammograms recorded in 5 mmol L^–1^ [Fe­(CN)_6_]^3–/4–^ and 0.1 mol L^–1^ KCl for each functionalization stage, showing a decrease in redox current and an increased peak-to-peak separation after antibody and antigen binding. (C) Nyquist plots from EIS measurements obtained under the same electrolyte, illustrating the progressive enlargement of the semicircular region and the corresponding rise in R_ct_ with each modification step. (D) Selectivity analysis of anti-hu IFN-α functionalized MN electrodes exposed to different test conditions (IL-17, IL-6, BSA, TNF-α, and IFN-α). The marked increase in R_ct_ for IFN-α demonstrates the high specificity of the biosensor toward its target cytokine.

Complementary electrochemical characterizations were performed using cyclic voltammetry (Figure 5B) and electrochemical impedance spectroscopy (Figure 5C), with [Fe­(CN)_6_]^3–/4–^ as the redox probe under standard conditions (0.1 mol·L^–1^ KCl). The CV curves (Figure 5B) showed decreased peak currents and increased peak separation after antibody–antigen binding, consistent with reduced charge-transfer kinetics due to biomolecular layer formation. EIS measurements further confirmed this trend, showing an increase in R_ct_ after each functionalization step (Figure 5C), indicating hindered diffusion of the redox probe caused by the formation of insulating biointerfaces.

Quantitative analysis of the Nyquist plots (Figure 5C) revealed a stepwise increase in R_ct_ during the successive stages of electrode biofunctionalization. The bare microneedles exhibited an R_ct_ of 911 ± 175 Ω, which increased significantly to 3298 ± 181 Ω after antibody immobilization, confirming the successful attachment of anti-hu IFN-α on the conductive PPy surface. To further evaluate the biosensor specificity, impedance measurements were conducted after exposure to various proteins (Figure 5D). Incubation with nontarget species such as TNF-α, IL-6, IL-17, and BSA resulted in negligible changes in R_ct_, whereas exposure to the target IFN-α antigen produced a pronounced increase to 8634 ± 382 Ω. This clear discrimination between target and nontarget responses, combined with the low variability among replicates, demonstrates the high specificity and reproducibility of the developed microneedle-based biosensor.

The influence of the PPy content in the composite was systematically investigated (Figure S8), as it governs both the electrical and morphological characteristics of the sensing interface. Among the evaluated formulations, MNs fabricated with PCL/PPy at 50 mmol·L^–1^ showed the highest ΔR_ct_ values upon antibody and antigen immobilization, along with low standard deviations across replicates, confirming superior sensitivity and reproducibility. In contrast, MNs containing either lower (10 and 25 mmol·L^–1^) or higher (100 and 200 mmol·L^–1^) PPy concentrations exhibited suboptimal performance with hardly any change in R_ct_, likely due to inefficient electron transfer or phase segregation effects.

At low PPy content, the conductive network may be insufficient to support reliable charge transport, leading to signal instability. Conversely, excessive PPy concentrations may result in structural heterogeneity and disrupted percolation pathways, thereby compromising interfacial electron transfer. These findings suggest that a balanced microstructure, achievable at 50 mmol·L^–1^ PPy, is crucial for maximizing biosensor performance, providing an optimal interplay between electrical conductivity and surface homogeneity.

Figure 6 presents the analytical performance of the microneedle biosensor for IFN-α detection, highlighting both response time and sensitivity. Panel 6A shows the variation of ΔR_ct_ as a function of incubation time between IFN-α (1000 pg·mL^–1^) and PCL/PPy (50 mmol·L^–1^)/Ab-functionalized electrodes. The ΔR_ct_ increased gradually from 5 to 20 min, indicating ongoing antigen–antibody interaction, and reached a stable plateau after 30 min. This behavior demonstrates that a 30 min incubation ensures a reliable and reproducible electrochemical response, defining the minimum response time for the biosensing protocol.

**Figure 6:**
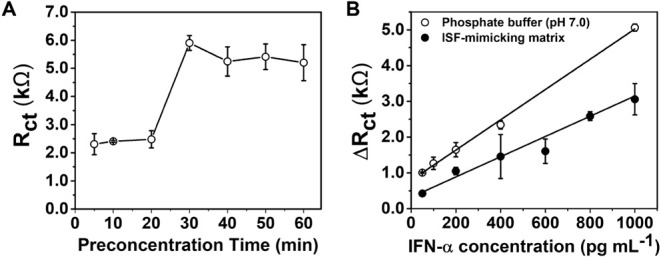
(A) ΔR_ct_ values obtained from EIS as a function of incubation time between the PCL/PPy 50 mmol·L^–1^/Ab microneedle biosensor and IFN-α (1000 pg·mL^–1^), showing signal stabilization after 30 min. (B) Calibration curves constructed from EIS data across IFN-α concentrations (10–1000 pg·mL^–1^) in phosphate buffer and synthetic interstitial fluid, showing strong linear correlation and minimal performance loss under physiologically relevant conditions.

In Figure 6B, calibration curves were constructed using EIS measurements across IFN-α concentrations ranging from 10 to 1000 pg·mL^–1^, in both phosphate buffer and a synthetic interstitial fluid containing HEPES (pH 7.4), NaCl, KCl, NaH_2_PO4, Sacarose, CaCl_2_, and MgSO_4_.^42^ A strong linear correlation was observed between the logarithm of IFN-α concentration and ΔR_ct_ in both media, with R^2^ values exceeding 0.98. The calculated limit of detection (LOD) was 8.66 pg·mL^–1^ in phosphate buffer and 12.75 pg·mL^–1^ in ISF, while the limit of quantification (LOQ) was 28.87 pg·mL^–1^ and 42.51 pg·mL^–1^, respectively. Despite the greater complexity of the ISF-mimicking medium, the biosensor maintained high sensitivity and reproducibility, confirming its robustness under physiologically relevant conditions.

A comparison with commercial ELISA kits (Figure S9), which have a reported detection range of 12.5 to 500 pg·mL^–1^ (extendable to 5000 pg·mL^–1^), highlights the advantages of the PCL/PPy 50 mmol·L^–1^ biosensor. Despite a slightly higher LOD compared to the ELISA assay (8.66 pg·mL^–1^ vs 5 pg·mL^–1^), the electrochemical platform offers significant benefits, including shorter analysis time (∼30 min vs over 3 h), reduced reagent consumption, and lower operational costs. This enhanced performance is attributed to the high surface area provided by the PCL/PPy matrix, which facilitates efficient recognition and quantification of IFN-α. These results reinforce the potential of this electrochemical microneedle platform for cost-effective and rapid cytokine detection in biomedical applications.

To assess the selectivity and sensitivity of the biosensor under more complex biological conditions, experiments were performed using porcine gelatin as a model matrix,^39,42^ either in its pure form or enriched with IFN-α at a concentration of 1 ng·mL^–1^ (Figure S10). Since the system replicates the principles of a commercial ELISA test, no blocking agents were used to minimize potential nonspecific interactions. Even without these agents, the electrochemical response remained largely unaffected by the gelatin matrix, as evidenced by the minimal signal variation observed in Figure S10A–C (green signal). Low electrochemical signals were also recorded in the absence of the anti-IFN-α antibody (yellow signal) or IFN-α antigen (orange signal), demonstrating that nonspecific interactions did not significantly influence the measurements.

When the full system was tested, PCL/PPy 50 mmol·L^–1^ sensitized with anti-IFN-α and incubated with IFN-α-enriched gelatin (Figure S10A–C, red signal), a 6-fold increase in the electrochemical response was observed, closely matching the results obtained in buffer-based analyses. This confirms that the biorecognition and immunocapture process remained effective even under simulated ISF conditions.

To further validate the biosensor’s performance under conditions that better replicate physiological diffusion, additional experiments were conducted using a Strat-M® artificial membrane in contact with a phosphate buffer solution containing IFN-α, mimicking a Franz diffusion cell configuration (Figure S10D). The membrane acts as a synthetic barrier with permeability and diffusional resistance similar to those of human skin, enabling the in vitro assessment of cytokine transport. The microneedle biosensor successfully detected IFN-α across the Strat-M® barrier, showing an increased R_ct_ response (blue signal) consistent with antigen recognition through the diffusion layer. These results confirm the device’s capability to operate under skin-mimicking diffusion conditions and reinforce its potential for in vitro interstitial fluid monitoring.

The comparison between the biosensor and conventional ELISA kits is summarized in Table S1. The electrochemical platform exhibited a comparable limit of detection (8.66 pg·mL^–1^ in buffer and 12.75 pg·mL^–1^ in synthetic interstitial fluid) to that of ELISA (LOD ≈ 5 pg·mL^–1^), but with significant practical advantages. While ELISA requires approximately 3 h and 15 min for the entire assay, the electrochemical method completes detection in 40 min, with minimal sample volume and without enzymatic labeling or multiple washing steps. Furthermore, the fabrication process is compatible with scalable and low-cost production, and the biosensor operates reliably in both buffered solutions and simulated interstitial fluid. These attributes position the PCL/PPy microneedle-based sensor as a robust, rapid, and cost-effective platform for point-of-care cytokine monitoring, with strong potential for clinical and wearable biomedical applications.

To further validate the robustness of the platform, additional experiments were performed to assess the time stability and fabrication reproducibility of the microneedle electrodes (Figure S11). The PCL/PPy 50 mmol L^–1^/anti-hu IFN-α biosensor exhibited a stable R_ct_ response during the initial measurements but showed a gradual decrease after 1 week, even when stored under refrigeration, indicating partial loss of biofunctional activity. Conversely, the independently prepared PCL/PPy (50 mmol L^–1^) microneedle electrodes displayed highly reproducible impedance spectra, with an average R_ct_ of 851.2 ± 31.1 Ω, confirming the consistency of the fabrication and polymerization processes.

In addition, the analytical performance of the biosensor was verified under realistic biological conditions through a recovery study using human plasma diluted 1:50 (Table S2). The table summarizes the average R_ct_ values, recovery, and relative standard deviation (RSD) obtained for different concentrations of IFN-α added to plasma samples from healthy donors (CAAE: 43139921.2.0000.5594). Recovery values ranged from 90.33% to 125.46%, indicating satisfactory analytical performance across most of the tested concentrations. For higher IFN-α concentrations (1000 and 800 pg mL^–1^), recoveries were close to 100%, indicating a reliable sensor response in this range. The overestimated recovery observed at 600 pg·mL^–1^ (125.46%) may be attributed to matrix effects caused by the nonspecific adsorption of plasma biomolecules onto the electrode surface. Nonetheless, all RSD values remained below 2.5%, confirming the high reproducibility of the electrochemical measurements. Overall, these results demonstrate that the biosensor exhibits good accuracy, precision, and recovery, supporting its applicability in analyzing complex biological matrices.

To further contextualize the analytical performance of the developed platform, Table S3 summarizes recent reports of electrochemical cytokine biosensors, including microneedle- and planar-based architectures, along with their main analytical parameters (LOD, linear range, and detection mode). Reported microneedle sensors for IL-6 typically achieve detection limits of ∼0.5 pg·mL^–1^, while planar IFN-γ sensors can reach subpg·mL^–1^ sensitivity. Despite these differences, the present PCL/PPy microneedle biosensor achieves comparable performance, with LODs of 8.66 pg·mL^–1^ in buffer and 12.75 pg·mL^–1^ in synthetic ISF, while operating via a label-free, minimally invasive offline detection workflow.

Moreover, the working concentration range and calibration parameters of the biosensor are fully consistent with those of the commercial ELISA kit used for antibody functionalization and method validation (VeriKine Human IFN-α ELISA Kit, Figure S9; assay range 12.5–500 pg·mL^–1^, extended range 156–5000 pg·mL^–1^). This alignment confirms that the electrochemical platform operates within a clinically relevant, standardized detection window while reducing the total assay time from over 3 h (for ELISA) to approximately 40 min under label-free conditions. Collectively, these results position the proposed PCL/PPy microneedle biosensor as a robust, scalable alternative to conventional immunoassays, offering rapid response, portability, and potential integration into point-of-care diagnostic systems.

### 3.6 Cytotoxicity Evaluation of PCL/PPy Microneedles

Cell viability assays conducted on two cell lines confirmed that the PCL/PPy microneedles exhibit excellent biocompatibility, with no significant cytotoxicity observed at the tested PPy concentrations (Figure S12). The only significant reduction in cell viability occurred in the positive control group (SDS-treated cells), thereby validating the assay’s sensitivity to cytotoxic responses. Both the untreated control group and all PCL/PPy formulations (10–200 mmol·L^–1^ PPy) maintained high cell viability, comparable to that of pure PCL microneedles.

These results indicate that PPy incorporation does not compromise cell survival, suggesting that its inclusion in the polymer matrix does not lead to the release of toxic degradation products or to undesirable interactions with the cellular environment.^47^ Although slight variations in cell viability were observed across different PPy concentrations, no dose-dependent cytotoxicity trend was evident. This supports the stability and safety of the composite material.

The consistently high cell viability observed in both immortalized human keratinocyte (HaCaT) and mouse embryonic fibroblast (3T3) cell lines confirms the biocompatibility of the PCL/PPy microneedles and reinforces their potential for biomedical applications, including biosensing, drug delivery, and tissue engineering.

## 4 Conclusion

This study presents the development of a minimally invasive microneedle-based electrochemical biosensor for IFN-α detection, achieving a favorable balance between sensitivity, specificity, biocompatibility, and structural robustness. Structural and morphological analyses confirmed the homogeneous incorporation of polypyrrole into the PCL matrix, with PPy content directly influencing thermal stability, surface roughness, and phase distribution. Raman spectroscopy and XRD measurements further validated structural modifications that enhanced electrochemical performance.

Among the tested formulations, a PPy concentration of 50 mmol·L^–1^ provided the optimal electrochemical response, combining enhanced charge-transfer kinetics with mechanical integrity and reproducibility. Biocompatibility assays confirmed the nontoxic nature of the platform, reinforcing its suitability for biomedical applications.

The biosensor demonstrated excellent analytical performance for IFN-α detection, with detection limits comparable to those of commercial ELISA assays, while offering additional advantages, including shorter analysis time, reduced reagent consumption, and label-free detection. The integration of a preconcentration step and the use of electrochemical impedance spectroscopy enabled reliable quantification in both buffer and complex synthetic interstitial fluids. Notably, the biosensor design supports an extraction plus offline analysis workflow, decoupling the sensing and readout stages. This configuration minimizes mechanical interference, eliminates the need for continuous skin contact, and simplifies integration with portable analytical devices—highlighting its potential for decentralized diagnostics.

Altogether, these findings establish the PCL/PPy microneedle biosensor as a cost-effective, scalable, and versatile platform for real-time cytokine monitoring, with promising implications for personalized medicine, immunotherapy management, and infectious disease diagnostics.

## Supplementary Material



## Notes

Additional figures (photographs of microneedle arrays; morphological, thermal, and electrochemical analyses including RMSD, TGA/DTG, EIS, and CV data); selectivity and diffusion tests using porcine gelatin and Strat-M® membrane; time-stability and reproducibility assessments; cell viability assays; and comparison tables of biosensor performance, recovery tests in plasma, and literature benchmarking of cytokine sensors (PDF)
