# Supplementary material for: Polypyrrole-Coated Microneedle Platform for Offline Electrochemical Detection of Interferon-Alpha in Interstitial Fluid
Source: ACS Appl Bio Mater. 2026 Feb 11;9(5):2419–33. doi: 10.1021/acsabm.5c01937 (PMC12958329; doi:10.1021/acsabm.5c01937)
Supplement: Supplementary file 1 [file mt5c01937_si_001.pdf]

Supporting Information

Polypyrrole-Coated Microneedle Platform for Offline Electrochemical Detection of Interferon-Alpha in Interstitial Fluid

Ana Carola Delavia Reis,[1] Ana Cristina Honorato de Castro-Kochi,[1,2] Jose Eduardo Ulloa Rojas,[1] Dylan A. Chiaro,[3] Suchismita Guha,[3] Gavin M. King,[3] Vivian L. Oliveira,[4] Daniele Ribeiro de Araujo,[5] Giovana Radomille Tofoli,[6] Mariano Romero,[7] Dominique Mombrú,[7] Wendel A. Alves.[1,*]

[1]Center for Natural and Human Sciences, Federal University of ABC, Santo André, SP 09210-580, Brazil.

[2]School of Biomedical Engineering, Einstein Hospital Israelita, São Paulo, SP 05521-200, Brazil.

[3]Department of Physics and Astronomy, University of Missouri, Columbia, MO 6521, USA.

[4]Laboratory of Immunology, INCOR, HCFMUSP Clinical Hospital, Faculty of Medicine, University of São Paulo, São Paulo, SP 05403-900, Brazil.

[5]Biophysics Department, Federal University of São Paulo, São Paulo, SP 04021-001, Brazil.

[6]Sao Leopoldo Mandic Faculty, São Leopoldo Mandic Research Institute, Campinas, SP 01332-000, Brazil.

[7]Faculty of Chemistry, University of the Republic, Montevideo 11800, Uruguay.

**Corresponding author:*

Wendel A. Alves (Federal University of ABC): wendel.alves@ufabc.edu.br

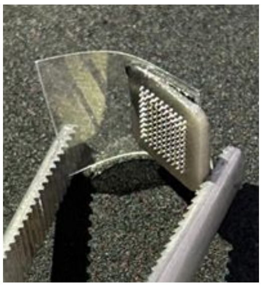

Figure S1. Photograph of a microneedle array integrated with an ITO/PET conductive film, demonstrating the physical configuration of the device for electrochemical applications.

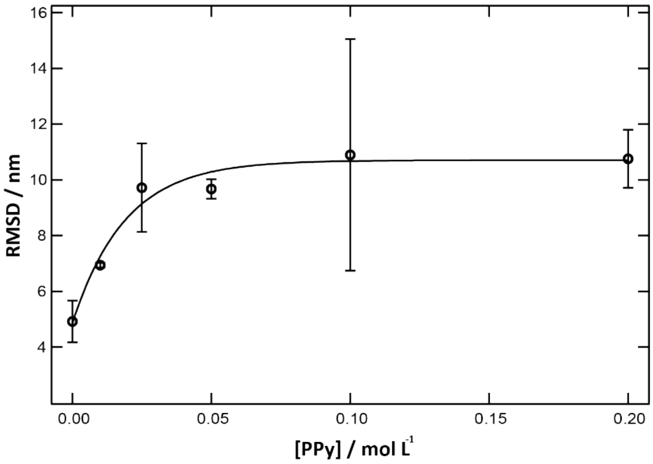

Figure S2. Quantitative analysis of RMSD as a function of PPy concentration in PCL/PPy microneedles. The error bars are the standard error of the mean, s/$\sqrt{N}$.

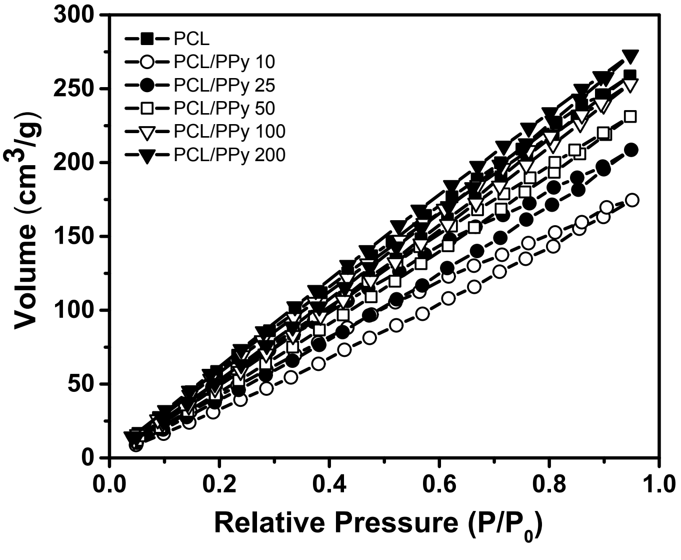

Figure S3. Nitrogen adsorption–desorption isotherms of pristine PCL and PCL/PPy microneedle samples obtained at 77 K.

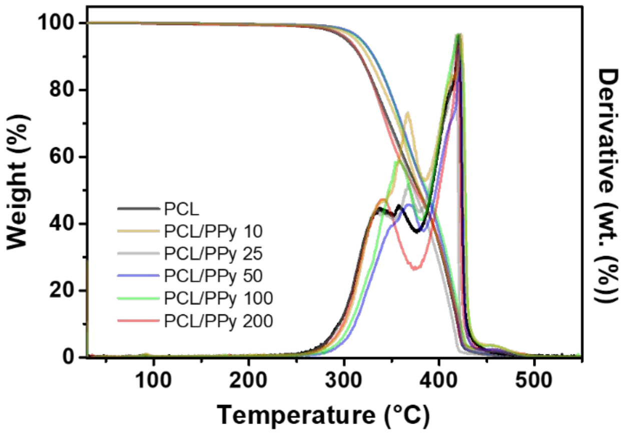

Figure S4. TGA and DTG of PCL and PCL/PPy MNs with different PPy concentrations.

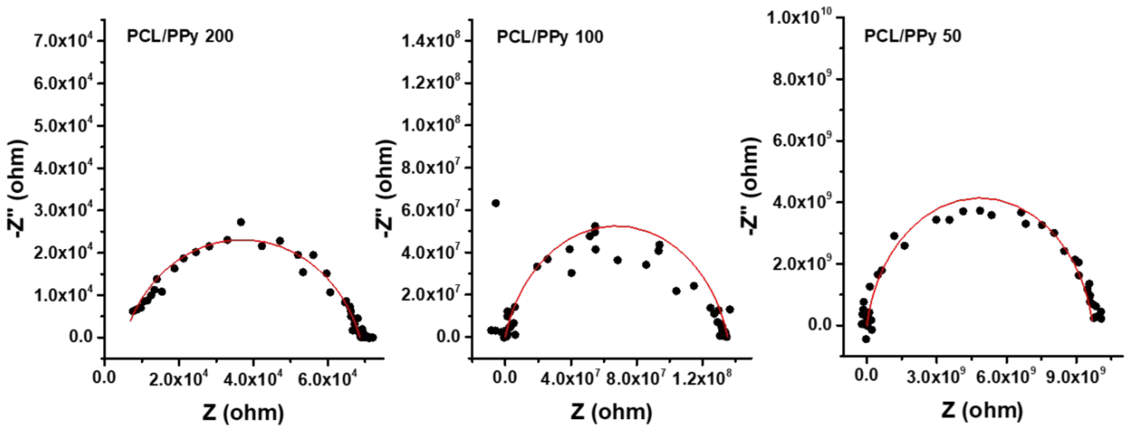

Figure S5. EIS measurements of PCL and PCL/PPy MNs in solid-state conditions.

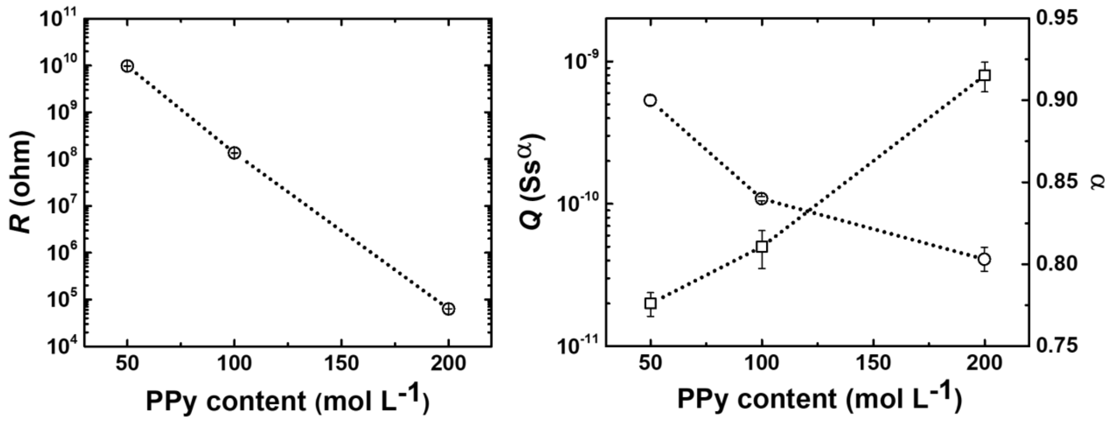

Figure S6. Correlation parameters extracted from EIS data of PCL and PCL/PPy MNs, show the influence of PPy concentration on charge transfer resistance and capacitive behavior.

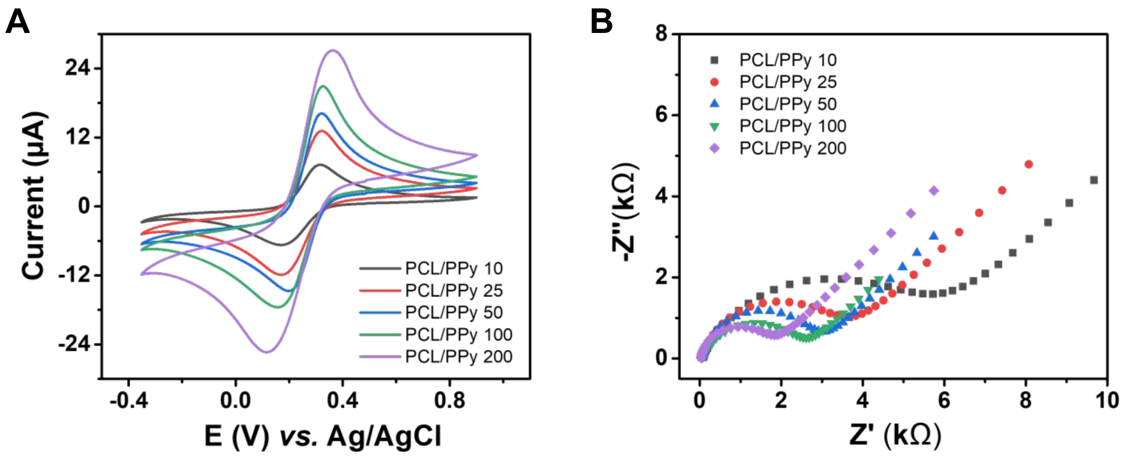

Figure S7. CV and EIS of PCL and PCL/PPy microneedles in 0.1 mol L^{-1} KCl solution, showing the influence of PPy concentration on charge transfer behavior and electrochemical activity.

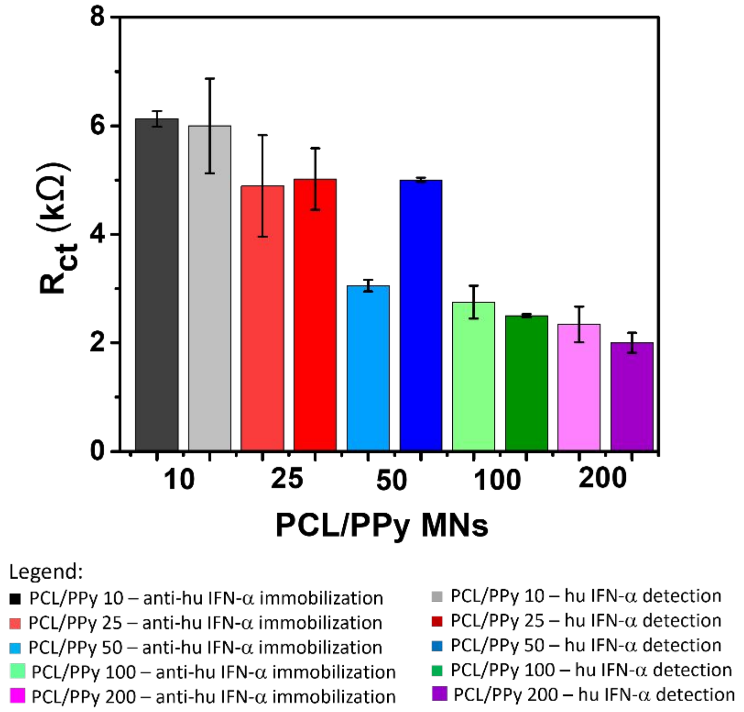

Figure S8. EIS data recorded for MNs fabricated with varying concentrations of PPy: 10, 25, 50, 100, and 200 mmol·L^{-1}. The Nyquist plots were obtained after functionalization with anti-IFN-α antibodies followed by incubation with the target analyte (IFN-α). The results indicate a maximum ΔR_{ct} for MNs prepared with 50 mmol·L^{-1} of PPy, evidencing optimized sensitivity and reproducibility at this composition.

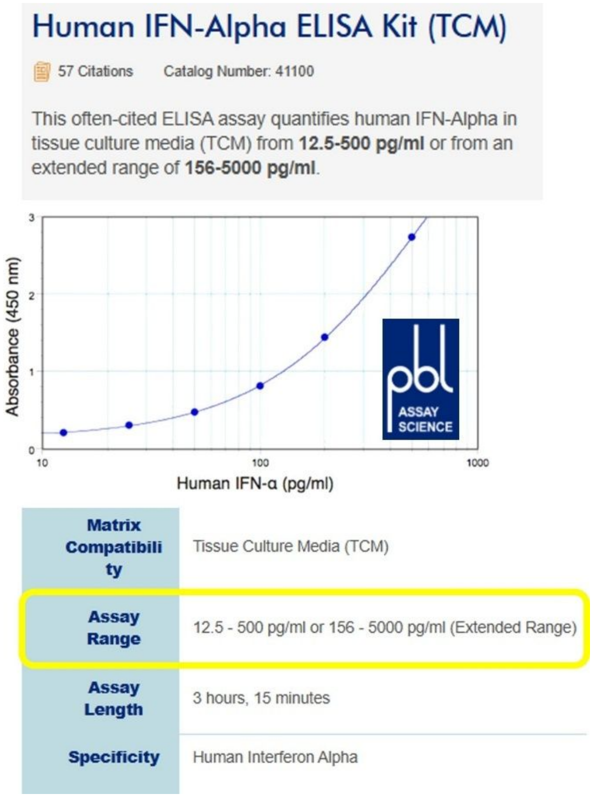

Figure S9. Technical specifications of the commercial Human IFN-Alpha ELISA Kit (PBL Assay Science, Cat. No. 41100), used for comparison purposes.

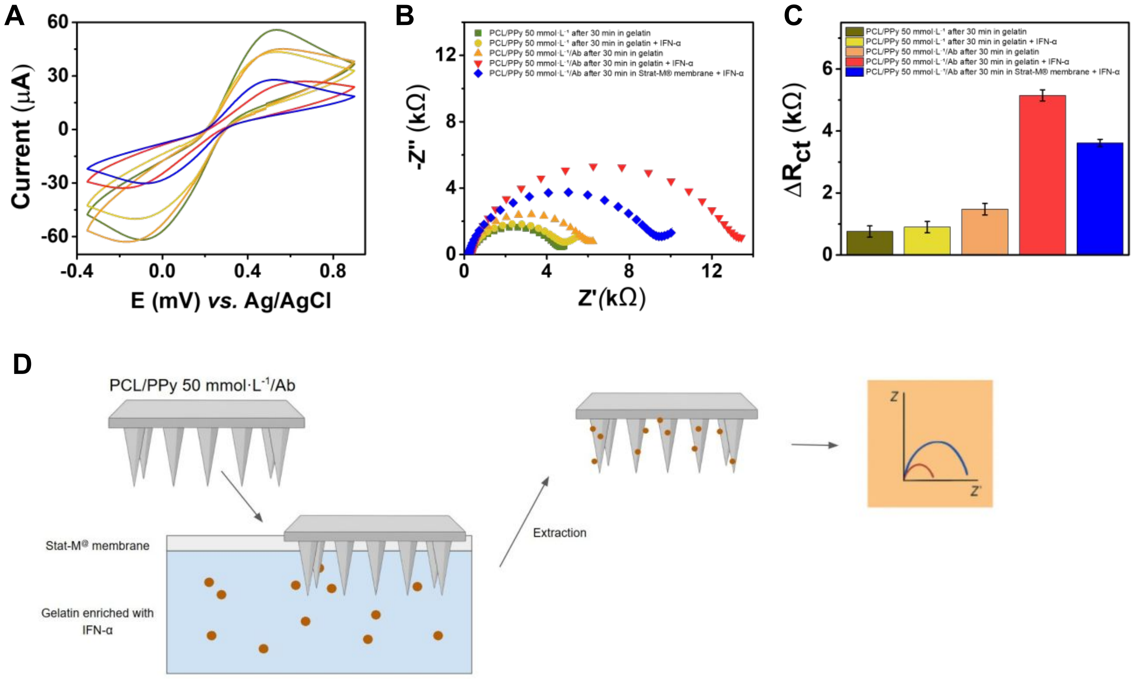

Figure S10. Selectivity and diffusion study under biologically relevant conditions using porcine gelatin and a Strat-M® artificial membrane as a skin-mimicking barrier. (A) Cyclic voltammograms, (B) Nyquist plots, (C) bar graphs derived from Randles circuit fitting, and (D) schematic representation of the experimental setup showing the microneedle biosensor applied over the Strat-M® membrane in contact with a phosphate buffer solution containing IFN-α, mimicking a Franz diffusion cell configuration. Green: PCL/PPy (50 mmol L^{-1}) after 30 min of

preconcentration in gelatin; Yellow: PCL/PPy (50 mmol L^{-1}) after 30 min of preconcentration in gelatin enriched with IFN-α; Orange: PCL/PPy (50 mmol L^{-1})/Ab after 30 min of preconcentration in gelatin; Red: PCL/PPy (50 mmol L^{-1})/Ab after 30 min of preconcentration in gelatin enriched with IFN-α; Blue: PCL/PPy (50 mmol L^{-1})/Ab in contact with a Strat-M® membrane over gelatin enriched with IFN-α, simulating transdermal diffusion through an artificial skin barrier. Electrochemical analyses were performed in 5 mmol L^{-1} $[Fe(CN)_6]^{3-/4-}$ containing 0.1 mol L^{-1} KCl. The increased R_{ct} observed for the blue dataset confirms successful IFN-α recognition across the Strat-M® barrier, demonstrating the biosensor's ability to detect cytokines under diffusion-limited, skin-mimicking conditions.

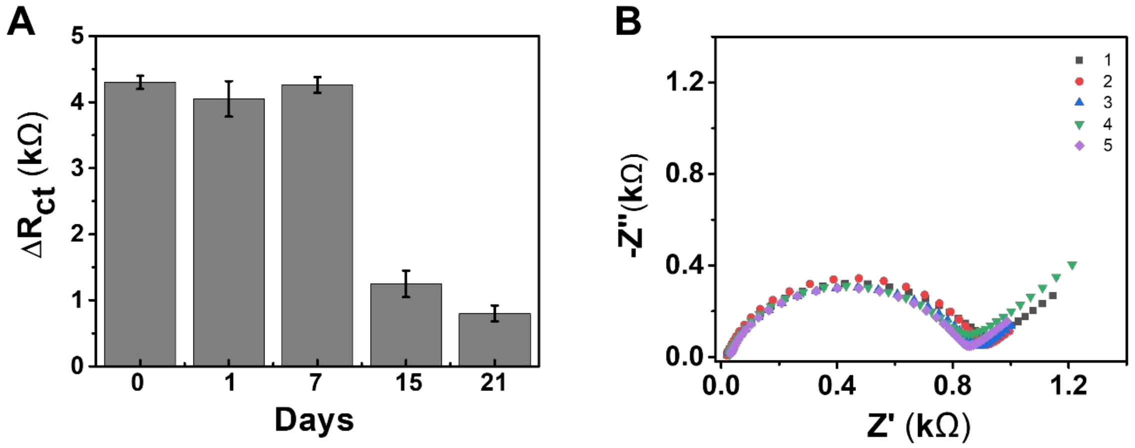

Figure S11. (A) Time-stability of the PCL/PPy 50 mmol L^{-1}/anti-hu IFN-α microneedle biosensor toward IFN-α (1000 pg mL^{-1}). The device maintained a consistent R_{ct} response during the initial measurements, followed by a marked decrease after approximately one week, indicating a loss of biofunctional activity of the recognition layer. During the testing period, the electrodes were stored under refrigeration when not in use. (B) Fabrication reproducibility: Nyquist plots of independently prepared PCL/PPy 50 mmol L^{-1} microneedle electrodes show close overlap, confirming the high reproducibility of preparation and biofunctionalization, with an average resistance of 851.2 ± 31.1 Ω.

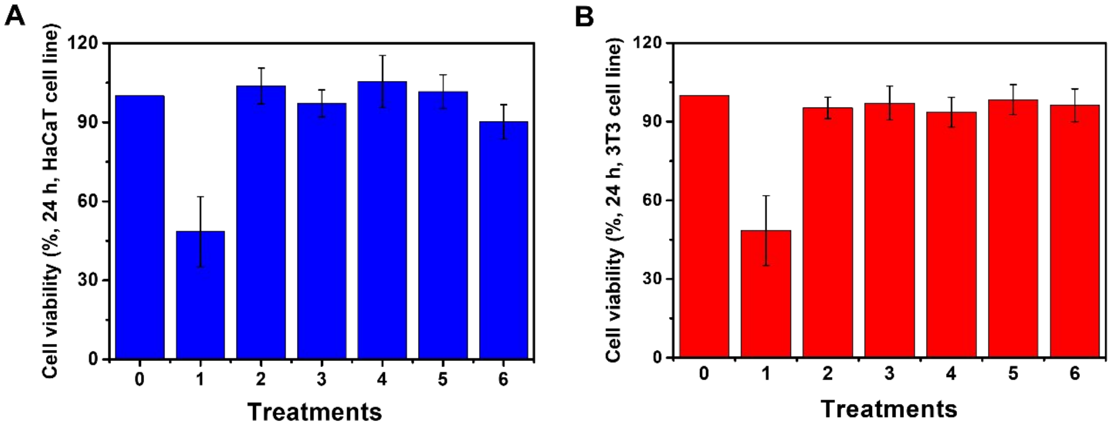

Figure S12. Cell viability of two cell lines exposed to PCL and PCL/PPy microneedles with varying PPy concentrations. (A) HaCaT keratinocytes and (B) 3T3 fibroblasts. The bars represent the relative viability percentage, where 0 corresponds to no treatment, 1 is the positive control (SDS-treated group), and 2–6 indicate increasing PPy concentrations (10, 25, 50, 100, and 200 mmol·L^{-1}). The results demonstrate that all PCL/PPy formulations maintained high cell viability, with no significant cytotoxic effects across the tested concentrations.

Table S1. Comparison of the analytical performance of the PCL/PPy microneedle-based electrochemical biosensor with conventional ELISA.

	**ELISA**	**Electrochemical (buffer)**	**Electrochemical (ISF)**
Assay range (pg mL^{-1})	12.5 - 1000	10 - 1000	10 - 1000
LOD (pg mL^{-1})	12.5	8.66	12.8
Assay length	3 hours, 15 minutes	40 min	40 min
Specificity	Human Interferon Alpha		

Table S2. Evaluation of the accuracy and precision of the microneedle biosensor for IFN-α detection in human blood plasma (1:50 dilution). R_{ct} values were obtained from Nyquist plots fitted to the Randles equivalent circuit. Electrolyte: 50 mmol L^{-1} $K_4Fe(CN)_6/K_3Fe(CN)_6$ in 0.1 mol L^{-1} KCl.

**Concentration of added antigen (pg mL^{-1})**	**R_{ct} (Average) (Ω)**	**Recovery (%)**	**RSD (%)**
400	1317	90.33	2.50
600	2015	125.5	1.40
800	2460	95.13	0.46
1000	3745	99.33	1.49

Table S3. Comparison of electrochemical biosensors for cytokine detection reported in the literature.

**Platform**	**Analyte**	**Detection mode**	**Sample matrix**	**LOD**	**Linear range**	**Remarks**	**Ref**
PCL/PPy MN + anti-IFN-α (this work)	IFN-α	EIS, label-free (R_{ct})	Buffer; Artificial ISF (gelatin)	8.66 pg·mL⁻¹ (buffer); 12.75 pg·mL⁻¹ (ISF)	10–1000 pg·mL⁻¹	Offline detection after immunocapture; readout in $[Fe(CN)_6]^{3-/4-}$; total assay time ≈ 40 min.	This work
Wearable MN (CNT–chitosan)	IL-6	EIS (immunosensor)	Artificial ISF; mouse model	0.54 pg·mL⁻¹	1–5000 pg·mL⁻¹	Continuous cytokine monitoring patch; early inflammation alert.	[1]
Si/Au MN immunosensor	IL-6	EIS (immunosensor)	Artificial ISF (gel matrix)	0.54 pg·mL⁻¹	1–5000 pg·mL⁻¹	Covalent antibody immobilization; stable performance for several days.	[2]
MN + MIP sensor	IL-6	EIS (molecularly imprinted polymer)	Artificial ISF	1.0 pg·mL⁻¹	1 pg·mL⁻¹ – 10 ng·mL⁻¹	Template-free recognition layer; robust and antibody-free.	[3]
Aptasensor (planar)	IFN-γ	EIS (aptamer)	10% human serum	0.67 pg·mL⁻¹	—	High sensitivity; non-MN planar configuration.	[4]
Immunosensor (planar)	IFN-γ	EIS (immunosensor)	Buffer	0.048 pg·mL⁻¹	0.1–10 000 pg·mL⁻¹	Ultra-low detection limit; planar electrode design.	[5]
Dual biosensor (planar)	TNF-α / IFN-γ	EIS (micro-gap)	Buffer	0.15 / 0.12 pg·mL⁻¹	—	Dual-target detection within 10 min.	[6]
Magnetic nanoparticle-assisted plasmonic biosensor	IFN-α	Surface plasmon resonance (optical)	Buffer; artificial plasma	102.5 pg·mL⁻¹	125–1250 pg·mL⁻¹	Metal-affinity magnetic nanoparticles coupled to a plasmonic surface; real-time, label-free detection within ~15 min; validated in artificial plasma.	[7]
General review examples	TNF-α (typical)	Various electrochemical modes	Saliva / ISF	1–30 pg·mL⁻¹	1–30 pg·mL⁻¹	Representative range for non-invasive cytokine sensors.	[8]

REFERENCES

[1] Xu, J.; Yang, B.; Kong, J.; Zhang, Y.; Fang, X. Real-Time Monitoring and Early Warning of a Cytokine Storm In Vivo Using a Wearable Noninvasive Skin Microneedle Patch. *Adv. Heal. Mater.* **2023**, *12*, 2203133. https://doi.org/10.1002/adhm.202203133.

[2] Russell, C.; Ward, A.C.; Vezza, V.; Hoskisson, P.; Alcorn, D.; Steenson, D.P.; Corrigan, D.K. Development of a Needle Shaped Microelectrode for Electrochemical Detection of the Sepsis Biomarker Interleukin-6 (IL-6) in Real Time. *Biosens. Bioelectron.* **2019**, *126*, 806–814. https://doi.org/10.1016/j.bios.2018.11.053.

[3] Oliveira, D.; Correia, B.P.; Sharma, S.; Moreira, F.T.C. Molecular Imprinted Polymers on Microneedle Arrays for Point of Care Transdermal Sampling and Sensing of Inflammatory Biomarkers. *ACS Omega* **2022**, *7*, 39039–39044. https://doi.org/10.1021/acsomega.2c04789.

[4] Noh, S.; Kim, J.; Park, C.; Min, J.; Lee, T. Fabrication of an Electrochemical Aptasensor Composed of Multifunctional DNA Three-Way Junction on Au Microgap Electrode for Interferon Gamma Detection in Human Serum. *Biomedicines* **2021**, *9*, 692. https://doi.org/10.3390/biomedicines9060692.

[5] Zhang, Y.; Zhang, B.; Ye, X.; Yan, Y.; Huang, L.; Jiang, Z.; Tan, S.; Cai, X. Electrochemical immunosensor for interferon-γ based on disposable ITO detector and HRP-antibody-conjugated nano gold as signal tag. *Mater. Sci. Eng., C* **2016**, *59*, 577–584. https://doi.org/10.1016/j.msec.2015.10.066.

[6] Seungwoo Noh, Hoseok Lee, Jinmyeong Kim, Hongje Jang, Jeongyun An, Chulwhan Park, Min-Ho Lee, Taek Lee. Rapid electrochemical dual-target biosensor composed of an Aptamer/MXene hybrid on Au microgap electrodes for cytokines detection. *Biosens. Bioelectron.* **2022**, *207*, 114159. https://doi.org/10.1016/j.bios.2022.114159.

[7] Saylan, Y.; Akgönüllü, S.; Denizli, A. Preparation of magnetic nanoparticles-assisted plasmonic biosensors with metal affinity for interferon-α detection. *Mater. Sci. Eng., B* **2022**, *280*, 115687.

[8] Lu, Y.; Zhou, Q.; Xu, L. Non-Invasive Electrochemical Biosensors for TNF-α Cytokines Detection in Body Fluids. *Front. Bioeng. Biotechnol.* **2021**, *9*, 701045. https://doi.org/10.3389/fbioe.2021.701045.
